# Potential of *Trichoderma harzianum* and Its Metabolites to Protect Wheat Seedlings against *Fusarium culmorum* and 2,4-D

**DOI:** 10.3390/ijms222313058

**Published:** 2021-12-02

**Authors:** Julia Mironenka, Sylwia Różalska, Przemysław Bernat

**Affiliations:** Department of Industrial Microbiology and Biotechnology, Institute of Microbiology, Biotechnology and Immunology, Faculty of Biology and Environmental Protection, University of Łódź, Banacha 12/16, 90-237 Łódź, Poland; julia.mironenka@edu.uni.lodz.pl (J.M.); sylwia.rozalska@biol.uni.lodz.pl (S.R.)

**Keywords:** plant elicitor, *Fusarium culmorum*, *Trichoderma harzianum*, proteomic study, antioxidative capacity

## Abstract

Wheat is a critically important crop. The application of fungi, such as *Trichoderma harzianum*, to protect and improve crop yields could become an alternative solution to synthetic chemicals. However, the interaction between the fungus and wheat in the presence of stress factors at the molecular level has not been fully elucidated. In the present work, we exposed germinating seeds of wheat (*Triticum aestivum*) to the plant pathogen *Fusarium culmorum* and the popular herbicide 2,4-dichlorophenoxyacetic acid (2,4-D) in the presence of *T. harzianum* or its extracellular metabolites. Then, the harvested roots and shoots were analyzed using spectrometry, 2D-PAGE, and MALDI-TOF/MS techniques. Although *F. culmorum* and 2,4-D were found to disturb seed germination and the chlorophyll content, *T. harzianum* partly alleviated these negative effects and reduced the synthesis of zearalenone by *F. culmorum*. Moreover, *T. harzianum* decreased the activity of oxidoreduction enzymes (CAT and SOD) and the contents of the oxylipins 9-Hode, 13-Hode, and 13-Hotre induced by stress factors. Under the influence of various growth conditions, changes were observed in over 40 proteins from the wheat roots. Higher volumes of proteins and enzymes performing oxidoreductive functions, such as catalase, ascorbate peroxidase, cytochrome C peroxidase, and Cu/Zn superoxide dismutase, were found in the *Fusarium*-inoculated and 2,4-D-treated wheat roots. Additionally, observation of the level of 12-oxo-phytodienoic acid reductase involved in the oxylipin signaling pathway in wheat showed an increase. *Trichoderma* and its metabolites present in the system leveled out the mentioned proteins to the control volumes. Among the 30 proteins examined in the shoots, the expression of the proteins involved in photosynthesis and oxidative stress response was found to be induced in the presence of the herbicide and the pathogen. In summary, these proteomic and metabolomic studies confirmed that the presence of *T. harzianum* results in the alleviation of oxidative stress in wheat induced by 2,4-D or *F. culmorum*.

## 1. Introduction

Nowadays, crops are exposed to numerous stress factors—natural ones, caused by the presence of pests and synthetic ones, related to the use of protective agents such as pesticides. One of the important problems of agriculture worldwide are soil borne diseases. The presence of pathogens and chemical stressors may affect the quality of crops and cause economic losses. Recently, biological control agents (BCAs) with reduced environmental impact have become an alternative to synthetic pesticides [1]. The innovative method of using bacteria and fungi to induce plant resistance to abiotic stress has been actively studied in recent years [2]. 

*Trichoderma* spp. are soil-inhabiting filamentous fungi, including species with antagonistic activity against plant pathogens, such as *Pythium* spp., *Fusarium* spp., and others. The process of the pathogen growth inhibition depends on various mechanisms, such as production of lytic enzymes and antibiotics and much faster space growth and nutrient consumption [3]. *Trichoderma* species are well known for their ability to produce various metabolites with antibiotic activity including peptaibols, polyketides, polypeptides, pyrones, and terpenes. 

*Fusarium* species are a group of dangerous cereal pathogens (FHB—*Fusarium* head blight) that produce metabolites belonging to important mycotoxins. The most toxic metabolites produced by these fungi include nivalenol and zearalenone (ZEA), which are harmful to animals and people [4]. 

In our previous work, the impacts of 14-aminoacids peptaibols and the metabolites T22-azophilone and harzianic acid in *T. harzianum* extracellular extract on *Fusarium culmorum* growth and development together with zearalenone production were studied [3]. After their ability to reduce *F. culmorum* growth and ZEA production had been found, it was decided to continue research using both *T. harzianum* and its metabolites to prevent the development of the pathogen in the plant–fungal system with wheat. 

According to our knowledge, *T. harzianum* can improve the germination of wheat kernels and reduce the toxic effects caused by the herbicide 2,4-D [5]. This strain is used as a commercial bioactive agent due to its ability to increase plant resistance against abiotic stresses and protect plants against pathogens [6] by competing with them for ingredients and even by parasitizing them [7].

Furthermore, 2,4D is a synthetic herbicide that mimics the natural hormone auxin, causing faster plant growth and leaf shedding. It was the first commercial herbicide to become popular because of its low cost, high efficacy, and selectivity. Crops such as wheat, rice, and barley are also susceptible to 2,4D’s negative effects. The changes may include twisting of the stems and leaves, deformation of the head, deformation of the roots, and inhibition of growth [8]. Additionally, 2,4-D may cause overproduction of reactive oxygen species (ROS) and lipid peroxidation [9]. 

Based on our previous research, in the present study we hypothesized that *T. harzianum* could be an effective agent by reducing different stresses exerted on plant germination. *F. culmorum* was chosen as a biological stress factor and 2,4-D as an additional chemical stress factor. To verify this hypothesis, the experiment was conducted with wheat (*Triticum aestivum*) as a model plant, as it is sensitive to 2,4-D and *F. culmorum*. Germinated wheat was exposed to *T. harzianum* and its extracellular metabolites, *F. culmorum* and 2,4-D. Multiple molecular biology techniques were used to assess the impacts of *T. harzianum* on the development of plants in the early stages of germination in the presence of stress factors, involving a proteomic study of the shoots and roots and analysis of the oxidoreductive enzymes’ activity and effects on jasmonic acid synthesis via ZEA determination.

## 2. Results and Discussion

### 2.1. Plant Germination and Growth Condition

In the present study, changes in the germination of wheat treated with different mixes of soil fungi with and without 2,4-D were investigated. To analyze how *Trichoderma* metabolites might act in a combined system of biological and chemical stress factors, *Trichoderma* metabolites extracts as well as *T. harzianum* fungus were used in the experiments. 

**Figure 1:**
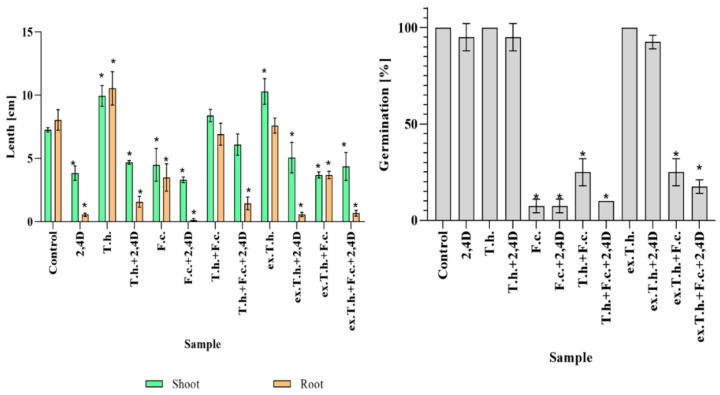
The total germination % values and the lengths of the seedlings measured after the 7th day of cultivation (T.h.—*T. harzianum*-inoculated wheat seeds; F.c.—*F. culmorum*-inoculated wheat seeds; ex.T.h.—*T. harzianum* extracellular metabolite-treated wheat seeds). Data represent means ± SE; the significance of the variance study with Tukey’s post hoc test = * *p* ≤ 0.001. The subcolumn statistic comparison is attached in supplementary material as Table S1.

To indicate changes taking place in the multivariate system, the first analysis was focused on plant germination and growth. The measurements were performed on the 7th day of cultivation (Figure 1).

The 2,4-D added to each of the tested systems had negative impacts on root development and shoots. When *T. harzianum* fungus was added, the growth of the stressed wheat roots improved. Both *Trichoderma* fungus and its metabolites had positive effects on wheat shoot length. *F. culmorum* added to the examined cultures inhibited the seeds development, while the presence of *T. harzianum* mitigated the negative impacts of the pathogen. The behavior of the systems after the addition of the herbicides and *Fusarium*, in which *Trichoderma* extract was used, was comparable with the control system. This might have been due to a limitation in the development of *Fusarium*, as had been previously observed on PDA plates [3].

In the present study, it was revealed that *F. culmorum* added to the wheat seeds had a huge impact on germination, causing at least a 70% inhibition. Similarly, Kaur et al. described wheat susceptibility to *Fusarium* infection. A decrease in winter wheat yield was noted in South Dakota, where seed germination and yield loss reached 80% [10].

*Trichoderma* species are well known biocontrol agents that promote plant growth. In the study by Bernat et al., the *Trichoderma* strain was able to reduce the impacts of herbicides on shoots and roots [5]. Usage of its metabolites alone in the presence of the herbicide was not sufficient for root development to a degree comparable to that caused by the fungus. 

### 2.2. Changes in Relative Water Content (RWC)

**Figure 2:**
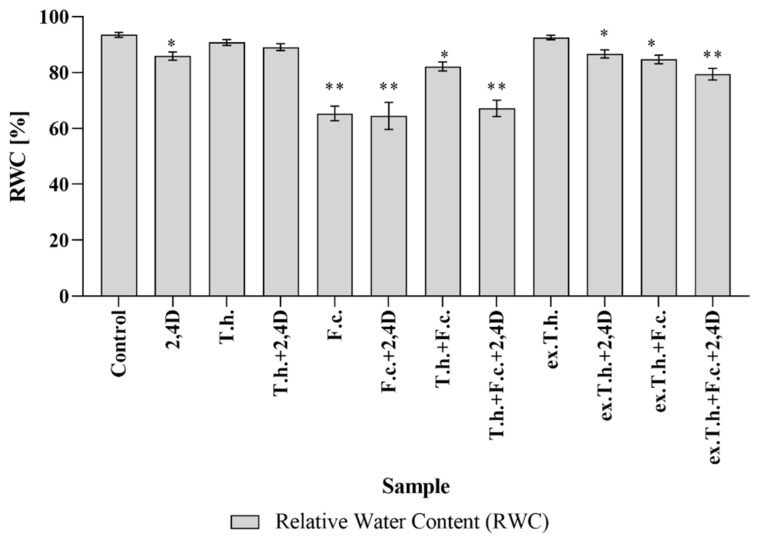
The RWC values of the shoot seedlings measured after 7 days of cultivation (T.h.—*T. harzianum*-inoculated wheat seeds; F.c.—*F. culmorum*-inoculated wheat seeds; ex.T.h.—*T. harzianum* extracellular metabolite-treated wheat seeds). Data represent means ± SE; the significance of the variance study with Tukey’s post hoc test = * *p* ≤ 0.001, ** *p* ≤ 0.0001. The subcolumn statistic comparison is attached in supplementary material as Table S2.

The energy status of a plant is determined by its water potential, which enables the transport of water in the soil–plant–atmosphere system [6]. In the present study, we decided to investigate how these stress factors would influence the basic parameters of wheat (Figure 2). In the absence of any wetting factors, the RWC in fully turgid transpiring leaves was found to reach above 98% and approx. 30–40% in wilting–dying or dried leaves [11].

*F. culmorum* added to the wheat culture significantly reduced the water content in shoots by up to 65% compared to uninfected control (~93%). Pshibytko et al. (2006), who studied tomato wilt caused by *Fusarium* species, revealed that the RWC decreased during *Fusarium* wilt development [12]. 

A lower value of RWC in the presence of the pathogen was noted, whereas *T. harzianum* and its metabolites contributed to an increase in the RWC value. The usage of *Trichoderma* extracts gave the best result in a system with chemical and biological stress factors. According to Mohapatra and Mittra, who studied the potential of *Trichoderma viride* to prevent the oxidative stress caused by *Fusarium oxysporum* in wheat, co-infected seedlings showed significant reinforcement of RWC values in comparison with *Fusarium*-infected seedlings [13].

### 2.3. Changes in Chlorophyll Content

The amount of chlorophyll present in the leaves depends on the nutritional status of the plants. It is known that a deficiency of some minerals disrupts the development of chloroplast pigments in general and chlorophyll in particular [14].

**Figure 3:**
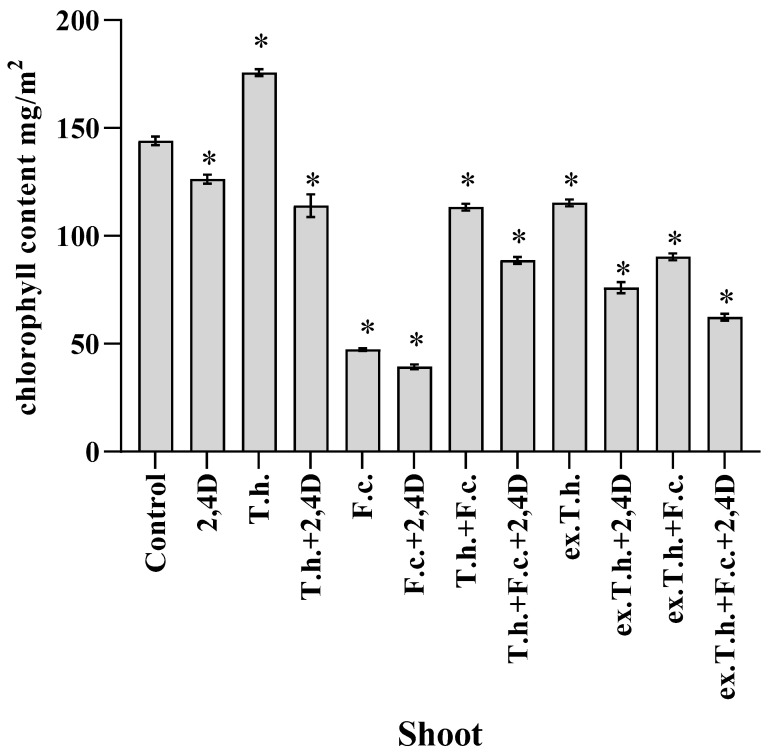
Chlorophyll contents in shoots measured after 7 days of cultivation (T.h.—*T. harzianum*-inoculated wheat seeds; F.c.—*F. culmorum*-inoculated wheat seeds; ex.T.h.—*T. harzianum* extracellular metabolite-treated wheat seeds). Data represent means ± SE; the significance of the variance study with Tukey’s post hoc test = * *p* ≤ 0.01. The subcolumn statistic comparison is attached in supplementary material as Table S3.

Martinez et al. [15], who studied the dependency between the concentration of chlorophyll and the leaf turgor in wheat, revealed that RWC did not cause differences in the chlorophyll content, which was also confirmed by the results obtained in the present study (Figure 3). 

Both the seedlings infected with *F. culmorum* and those treated with 2,4-D and the pathogen had the lowest concentrations (up to 50 mg/m^2^) of chlorophyll compared to the other wheat systems. Such reductions in chlorophyll content, which depend on the membrane stability, can be expected under stress conditions [16].

The seedlings infected by *Trichoderma* contained more chlorophyll. Even in contact with the studied stress factors, usage of the extract was not as effective. These results are in agreement with the findings of Zhang et al., who studied wheat grains infected with *T. longibrachiatum*, which contributed to the increased content of chlorophyll compared to the control system [17].

### 2.4. Changes in Jasmonic Acid 

Jasmonic acid is responsible for the development and regulation of diverse plant defense responses. The molecules are derived from cyclic fatty acids and synthesized in response to various stressors [18].

**Figure 4:**
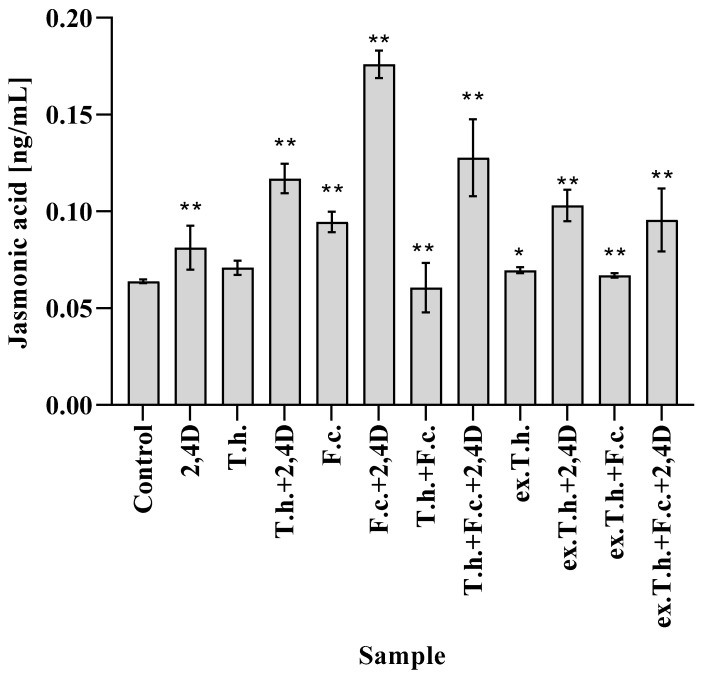
The contents of jasmonic acid in shoots measured after 7 days of cultivation (T.h.—*T. harzianum*-inoculated wheat seeds; F.c.—*F. culmorum*-inoculated wheat seeds; ex.T.h.—*T. harzianum* extracellular metabolite-treated wheat seeds). Data represent means ± SE; the significance of the variance study with Tukey’s post hoc test = * *p* ≤ 0.01, ** *p* ≤0.001. The subcolumn comparison is attached in supplementary material as Table S4.

During the analysis of early germinated shoots of wheat, different amounts of JA were noted (Figure 4). The pathogen inoculated on the seeds raised the content of the studied hormone compared to the control system. It is known that fungal infection is accompanied by changes in the amounts of two main hormones, salicylic acid (SA) and jasmonic acid, as well as the activation of SA and JA signaling [19].

Herbicide treatment of wheat resulted in only a slight lift in the level of the studied hormone. An approximately three-fold higher JA content (0.18 ng/mL) compared to the control system (0.06 ng/mL) was observed in the culture with the addition of both stress factors. The application of *Trichoderma* fungus reduced the hormone levels in the presence of *Fusarium*, although in the system with two stress factors the efficacy was very low. *T. harzianum* added to the wheat grains slightly increased the activity of JA, which is compatible with the findings of Moran-Diez et al., who studied the responses of tomato plants to the application of different *Trichoderma* species against *Pseudomonas syringae* [20].

It is known that increasing amounts of JA induce the expression of enzymes involved in the breakdown of chlorophyll [21]. There is a dependency between the amount of the studied hormone and the chlorophyll content (Figure 3)—the higher hormone concentration, the lower chlorophyll content. The exception was the system with *T. harzianum* and 2,4-D, in which a fairly high content of chlorophyll was noted. 

### 2.5. Changes in Oxylipins

Jasmonic acid is an oxylipin hormone that is crucial for plants to regulate growth and stress response [21]. Oxylipins are formed in an enzymatic way but are also produced in response to singlet oxygen or reactive forms [22]. Taking into consideration these changes of the hormone content, the level of oxylipin in the shoots was measured. The testing was also conducted on the roots, as they are a major site of microorganism–plant interactions. 

*Trichoderma* activity in the wheat system resulted in significant changes in the levels of the tested fatty acid derivatives in wheat shoots. In the presence of the herbicide, the levels of 9-Hode and 13-Hode derived from linoleic fatty acids were reduced. The metabolites of *T. harzianum* added to the seeds controlled the 13-Hotre level in the herbicide-treated system. A comparison of the cultures inoculated with *F. culmorum* showed significant growth of the oxylipin levels in shoots, which was effectively reduced by the presence of *T. harzianum* or its extracts. 

**Figure 5:**
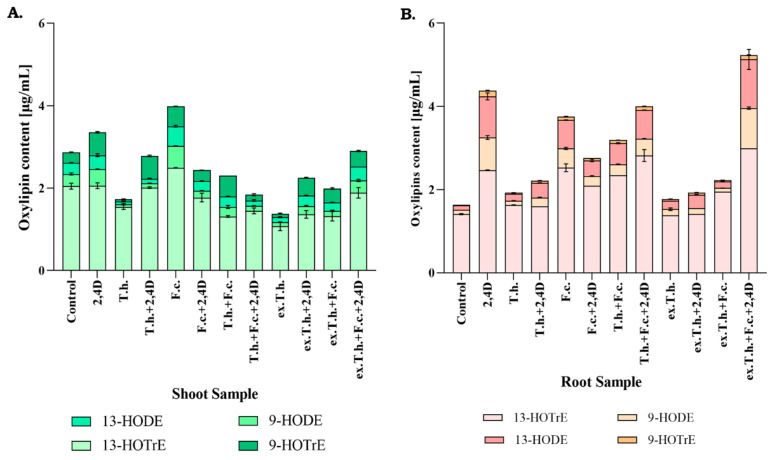
Relative oxylipin amounts in the shoots (**A**) and roots (**B**) measured after 7 days of cultivation (T.h.—*T. harzianum*-inoculated wheat seeds; F.c.—*F. culmorum*-inoculated wheat seeds; ex.T.h.—*T. harzianum* extracellular metabolite-treated wheat seeds). Data represent means ± SE; the significance of the variance study with Tukey’s post hoc test. The subcolumn comparison is attached in supplementary material as Table S5.

According to the obtained results (Figure 5), the presence of the herbicide induces the oxylipin levels in the root systems. Differences in 9-Hode and 13-Hode of linoleic origin were observed, while 13-Hotre from α-linolenic acid was also tracked. *F. culmorum* had the greatest impact on the derivatives of linoleic fatty acids. Seedlings exposed to *Trichoderma* or its metabolite activity demonstrated levels similar to the control system of the test oxylipins. In the presence of 2,4-D or the pathogen, these levels were reduced. It had been previously revealed by our team that *T. harzianum* diminished oxidative stress caused by 2,4-D [5]. The effectiveness in reducing the stress caused by the pathogen action has not been studied yet.

### 2.6. Differences in Antioxidant Enzyme Activity

It is known that JA induces reactive oxygen species (ROS), which damage chloroplasts in the first target and induce senescence [21]. Additionally, due to the changes in the oxylipin content, the activity levels of antioxidant enzymes in wheat shoots and roots were measured.

The mechanism of enzymatic decomposition of ROS proceeds in two steps—in the first step, superoxide dismutase is active, followed by the activation of catalase [22]. 

**Figure 6:**
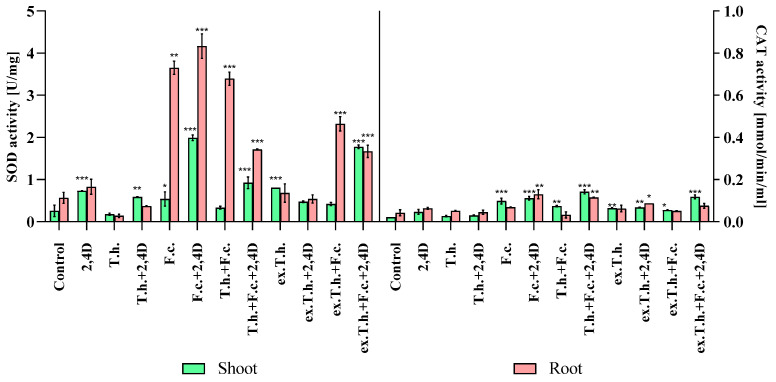
The oxidoreduction enzyme activity levels of the seedlings measured after 7 days of cultivation (T.h.—*T. harzianum*-inoculated wheat seeds; F.c.—*F. culmorum*-inoculated wheat seeds; ex.T.h.—*T. harzianum* extracellular metabolite-treated wheat seeds). Data represent means ± SE; the significance of the variance study with Tukey’s post hoc test = * *p* ≤ 0.05, ** *p* ≤ 0.01, *** *p* ≤ 0.001. The subcolumn statistic comparison is attached in supplementary material as Table S6.

Taking into consideration the fact that 7 days wheat seedlings were used in the study, the main focus was on the first response step (Figure 6). 

As the main interaction took place in the root system, the most pronounced changes were found in the enzyme activity in this part of the plant. The enzyme presence in the shoots might have been related to JA activity, causing the ROS induction. 

The presence of the herbicide stimulated the activity of superoxide dismutase (SOD) in the roots of all studied samples. *T. harzianum* decreased the SOD activity in the systems, whether treated or untreated with 2,4-D. The addition of *F. culmorum* to wheat seedlings resulted in extreme activation of the first-wave defense mechanisms against ROS. The highest activity (over 4 U/mg) was detected in the presence of chemical and biological stressors. In the presence of the studied biocontrol agent, the enzyme activity was lower, although it was not completely abolished.

The catalase (CAT) activity might not have revealed such significant differences because of the early stage of wheat development. Higher activity was noted in both plant organs in *Fusarium*-inoculated seeds in the presence of the herbicide. 

Interesting findings were reported by Tančić-Živanovet al., who studied the effects of *Trichoderma* spp. on the antioxidant activity and growth promotion of pepper seedlings [23]. Positive correlations were found between activity levels of SOD, CAT, and other enzymes and germination in plants treated with the studied isolates. It could be possible that the high activity levels of these enzymes in stress-exposed and *Trichoderma*-inoculated systems had an effect on the amount of energy needed for growth. On the other hand, the activity was lower compared to the *Fusarium*- or 2,4-D-treated seeds. Perhaps, the spore concentration or time of growth should have been chosen differently to ensure better antioxidant effectiveness of *T. harzianum*.

### 2.7. Changes in Zearalenone Content

Zearalenone is one of the most toxic mycotoxins, which is produced by *Fusarium* species and exerts deleterious effects on the reproductive capacity of animals [24]. 

**Figure 7:**
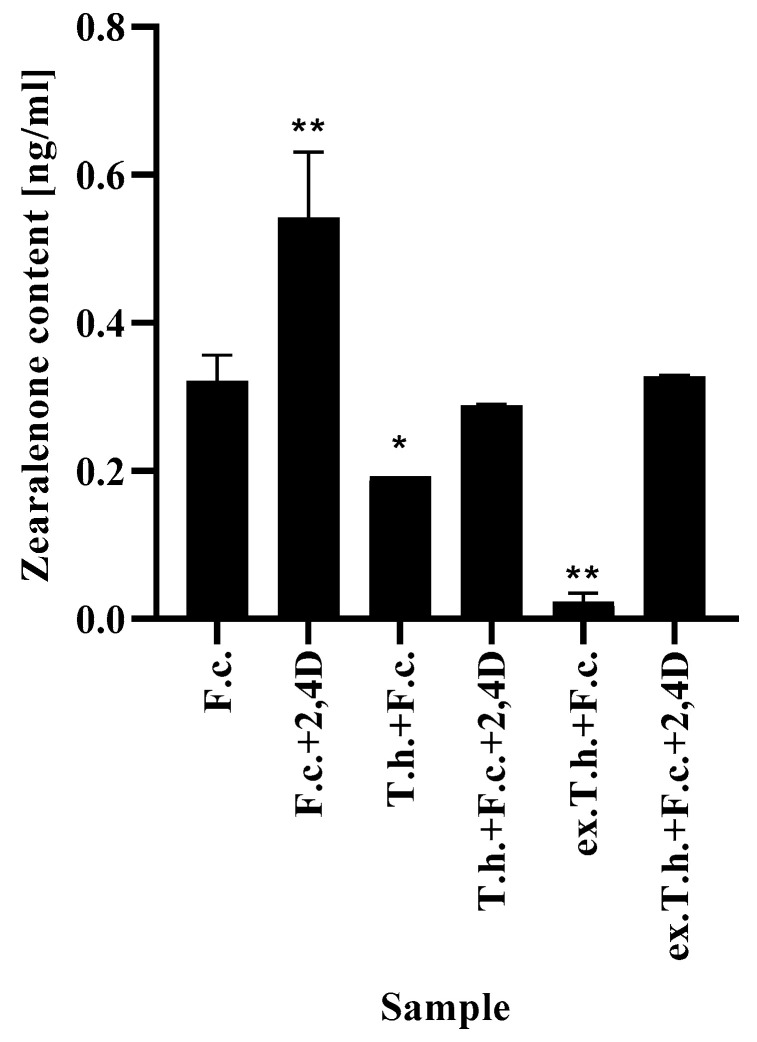
The ZEA levels in filter paper after 7 days of cultivation (T.h.—*T. harzianum*-inoculated wheat seeds; F.c.—*F. culmorum*-inoculated wheat seeds; ex.T.h.—*T. harzianum* extracellular metabolite-treated wheat seeds). Data represent means ± SE; the significance of the variance study with Tukey’s post hoc test = * *p* ≤ 0.01, ** *p* ≤ 0.001. The subcolumn statistic comparison is attached in supplementary material as Table S7.

In order to eliminate the development and toxic effect of *F. culmorum*, *T. harzianum* and its metabolites were added to the wheat culture. The results presented above (Figure 7) show the changes in mycotoxin production by the pathogen inoculated on the grains. Under standard conditions, the production of ZEA was approximately 0.3 ng/mL, while the treatment with 2,4-D raised the production capacity to 0.5 ng/mL, which was the highest possible concentration. The herbicide might have supported the development of the pathogen by limiting the plant growth. 

The presence of *T. harzianum* in the system with the pathogen resulted in weaker production of mycotoxin. It is known that for the effective production of mycotoxins, *Fusarium* species require nutrient availability [25]. The emergence of such a persistent competitor as *Trichoderma* limits the pathogen development and results in the activation of defense mechanisms. The most effective method turned out to be the addition of *T. harzianum* extracts, which were found to influence the development of the fungus [3]. This observation is consistent with the study by Gromadzka et al. [26], who showed that two various *Trichoderma* isolates reduced the amounts of ZEA produced by four different *Fusarium* strains in solid substrate bioassays.

Comparing the possible usage of *Trichoderma* or its metabolites, the second option is a much more effective method—from the very beginning the metabolites acted with *Fusarium*, interfering with the fungal development. The application of *T. harzianum* was less effective, because the fungi needed time to produce the metabolites externally, which gave a temporary opportunity for pathogen development.

### 2.8. Proteomic Study

There are two sensitive methods for protein analysis—MALDI TOF/TOF and LC-MS/MS. The first one with a very high processing capacity, the second, much more sensitive, capable of detecting several proteins in one spot [27].

The aim of poteomic study, included in present work, was not to interpret the entire protein map, but to look for the most pronounced changes that correlated with the previously obtained results.

Proteomic analysis based on estimation of the changes of proteins produced by the plant under different conditions was performed. The intensity values (spot volumes) were multiplied and spots with different volumes (the sum of the pixel intensities within the spot area) were excided out and digested for determination. A few spots were identified as being the same protein or representing isoforms. It is known that some spots contain several proteins, but analysis that was used in the present study have the lack in sensitivity compare to the LC-MS/MS [28], while the results given below show that the goal has been achieved, and our basic proteomics proved to be helpful in confirming the previously obtained results.

The results showed only the greatest changes detected in wheat shoots and roots (Table 1). SDS gels with marked spots are attached as Supplementary Figures S1–S4.

The study of the proteomic profile revealed changes in important processes of individual systems. The root system is the main place of the microorganism–plant interaction and the main area affected by the herbicide. 

The Table 1 shows results of the theoretical masses, accession number, scores, proteins ID obtained from the database search. The study of the results in the second part was based on selection of the data with the highest scores, comparison of their theoretical and experimental masses, definition of the protein function and its comparison to the earlier obtained results. It was found that some proteins differ in theoretical MW of from the proteins and mass obtained on the gel. It may be caused by several reasons: starting from the imperfections of gels due to the large numbers of treatment and trials, also the chemical modifications taking place in proteins may also have an impact on the changes [29].

According to UniProtKB specified functions, most proteins determined in our study were involved in stress response and antioxidation processes, such as oxidative stress response or superoxide metabolism, as well as in carbohydrate metabolism or glycolytic processes. Higher volumes of proteins and enzymes performing oxidoreductive functions, such as catalase, ascorbate peroxidase, cytochrome C peroxidase, and Cu/Zn superoxide dismutase, were found in the *Fusarium*-inoculated and 2,4-D-treated wheat roots. *Trichoderma* and its metabolites present in the system leveled out the mentioned proteins to the control volumes. These findings confirmed our earlier study on the enzyme activity levels.

Such enzymes as alpha amylase (theoretical MW 47,317 Da, SDS gel result ~46 kDa), malate dehydrogenase (theoretical MW 24,332 Da, in gel mass ~24 kDa), and S-adenosylmethionine synthetase (theoretical MW 43,153 Da, analysis result ~44 kDa) function in carbohydrate metabolism, while cathepsin, having catalytic activity, dominates in *T. harzianum*-inoculated systems and cultures treated with its metabolites. Smaller volumes of these spots were specified in the samples treated with the herbicide or inoculated with the pathogen. However, chitinase (theoretical MW 28,242 Da, result in gel ~27 kDa)—which degrades polymer chitin into low-molecular-weight particles [30]—dominated in the presence of *F. culmorum*, which is in line with Boller’s [31] findings that plant species may accumulate chitinases in response to plant pathogen infection. Due to this activity, chitinolytic enzymes have become some of the most promising candidates in the management of plant diseases [26]. 

An increased 12-oxo-phytodienoic acid reductase (theoretical MW 40,578 Da and in gel mas ~41 kDa) is involved in the oxylipin signaling pathway in wheat [32]. The activity of this protein was detected in herbicide-treated and *Fusarium*-inoculated wheat seeds. Additionally, there were significant changes in the oxylipin content in the roots of the analyzed samples. These results corroborate previously detected oxylipin levels. 

Four proteins are deemed to be involved in the glycolytic process, one of which- glyceraldehyde-3-phosphate dehydrogenase (GAPDH) predominates in the entire proteomic profile. GAPDH catalyzes glyceraldehyde-3-phosphate to 1,3-diphosphoglycerate. This step is accompanied by the binding of phosphate to triosis in the metabolism of inorganic glucose [33]. In this study, the highest amount of this protein was found in control and *Trichoderma*-inoculated wheat systems. The smaller intensity of this spot in the wheat system in which *F. culmorum* was present was replaced with a higher value of triosephosphate isomerase (TPI) (theoretical MW 26,765 Da, in gel ~26 kDa). The understanding of the function of the TPI protein in plant–pathogen interactions is still very limited [34]. 

Another protein that shows clear differences in intensity and exhibits ATPase activity is ATP synthase beta (theoretical MW 59,212 Da, in gel ~60 kDa) The inhibition of this enzyme compromises the output of ATP by oxidative phosphorylation [35]. In our study, the intensity of this protein spot was lower in all *Fusarium*-inoculated systems. 

Another large portion of proteins is involved in stress response and defense mechanisms, the value of which was shown to be predominant in the systems inoculated with the fungus. Chaperone proteins (theoretical MW 25,557 Da, in gel ~24 kDa) that assist the protein folding under stress conditions are essential for cell viability in all growth conditions [36]. Other germin-like proteins (GLPs) with plant response activity against abiotic and biotic stresses include plant glycoproteins belonging to the cupin superfamily [37]. The advantage of these proteins is that they may function as antioxidant enzymes, which explains their higher content in all *F. culmorum*-inoculated and 2,4-D-treated samples. 

Among the 30 studied proteins examined in the shoots, the proteins involved in photosynthesis and oxidative stress response attracted most of the attention. In this group, RUBISCO activase beta (theoretical MW 41,427 Da, in gel ~40 kDa), oxygen evolving enhancer (OEE) protein (theoretical MW 27,154 Da, in gel ~26 kDa), and ribulose 1,5 bisphosphate arboxylase predominated in *T. harzianum*-inoculated systems and in wheat shoots grown with *Trichoderma* extracellular metabolites. The lowest intensity of the spots was found in the presence of *Fusarium* or 2,4-D. The OOE proteins are essential for photosynthesis and are involved in the photo-oxidation of water during the light reactions [38]. The absence of the protein results in reduced rates of photosynthetic oxygen evolution [39]. Zadražnik et al. studied the drought stress effects on chloroplast proteins, with their findings describing the lower RWC linked to a lower amount of photosynthetic proteins [38]. Tracking our results, some dependence may be found between the lower RWC caused by chemical (2,4-D) or biological (*F. culmorum*) stress and the higher amount of photosynthetic proteins. 

Another large portion of the studied proteins responsible for glutathione metabolism was observed and the amounts of these proteins were higher in the herbicide-treated sample and in *F. culmorum*-inoculated wheat. Plant glutathione transferase (theoretical MW 24,984 Da, in gel ~24 kDa), an enzyme capable of catalyzing the conjugation and detoxification of herbicides, was found in higher amounts in all 2,4-D-treated cultures in this study [40]. 

Dehydroascorbate reductase (DHAR) (theoretical MW 23,334 Da, in gel ~23 kDa), an enzyme belonging to the glutathione S-transferase (GST) superfamily, reduces glutathione in the ascorbate–glutathione pathway, which is believed to play a key role in H2O2 metabolism [41]. Substantial growth of this protein was found in all *Fusarium*-treated cultures. Even the presence of *Trichoderma* or its extract did not significantly lower the values. Parween et al. revealed that the application of exogenous JA under pesticide treatment induces the biosynthesis of ascorbate, glutathione, phenols, and tocopherol, which plays an important role in ROS scavenging [42]. According to our results, it can be seen that the increase in the amount of intracellular JA (as a response to pathogen infection) and the presence of the herbicide resulted in higher contents of proteins with oxidoreductive function. 

Cyclophilins (theoretical MW 18,367 Da, in gel ~17 kDa) have been found to be versatile metabolites capable of regulating various processes in plant development and survival. They regulate multiple signaling and metabolic pathways and are able of binding such enzymes as thioredoxins and 2-Cys peroxiredoxin in chloroplasts [43]. In general, these proteins are present in different compartments of cells and are involved in various physiological processes. During our analysis, a higher content of these proteins was found in all systems with 2,4-D and in the *T. harzianum*-inoculated wheat system. Due to its ability for mRNA accumulation, S-adenosylmethionine synthetase (SAM) is an important protein of the defense mechanism in plants response to stress [44]. In our study, the largest spots of SAM were detected in all 2,4-D-treated cultures and *Trichoderma* metabolite-inoculated wheat seeds. 

## 3. Materials and Methods

### 3.1. Reagents

All chemicals used in the proteomic analysis were purchased from Hercules, (Hercules, CA, USA), Promega (trypsin) (Madison, WI, USA), and Sigma-Aldrich (St. Louis, MO, USA). The oxylipin standards were obtained from Cayman Chemicals (Ann Arbor, MI, USA), while zearalenone and aurofusarin standards came from Sigma-Aldrich. The other materials, including solvents and plastic labware, were obtained from Avantor Performance Materials (Gliwice, Poland) and Eppendorf (Hamburg, Germany).

### 3.2. Plant Materials and Growth Examination

*Triticum aestivum* L. seeds (cv. Zyta) (Łódź, Poland) were selected based on the results of our previous research [5]. The culture was conducted as described in our previous work, with slight modifications. A total of 20 pre-sterilized seeds were placed onto two layers of filter paper and inserted onto 5 cm petri dishes. Next, 3 mL of water (with or without the herbicide and fungal spores) was added. During the germination, 1 mL of distilled water was added per day. The conditions of the plant growth chamber (IL/750/FIT P Pol-Eko, Poland) were not changed in a 14 h light/10 h dark photoperiod, with light supplied by cool white fluorescent lamps (a light intensity of 200 µmol m^−2^ s^−1^) and the relative humidity being set to 40%. 

The samples included one independent control with distilled water, 2,4-D-treated culture, *T. harzianum* alone and with 2,4-D, *F. culmorum* and *F. culmorum* with 2,4-D, *T. harzianum* with *F. culmorum*, and *F. culmorum* with 2,4-D. The extract of *T. harzianum* secondary metabolites was also studied alone and with 2,4-D, with *F. culmorum*, and separately with *F. culmorum* and 2,4-D. The 12 groups were examined and the process was repeated three times. The lengths of the shoots and roots were measured after 7 days of cultivation. 

### 3.3. Fungal Inoculum

*Trichoderma harzianum* KKP534 from the fungal strain collection of the Department of Industrial Microbiology and Biotechnology, University of Lodz, and the strain *F. culmorum* DSM 1094 purchased from the German Collection of Microorganisms and Cell Cultures GmbH were used in the study. The strains were selected for the study based on our previous research [3], where *T. harzianum* extracellular metabolites caused the inhibition of *F. culmorum* growth and development. 

Spores isolated from 7 days cultures grown on ZT or Sabouraud agar slants were used in the study [3]. The final concentration of 3 × 10^8^ spores per Petri dish of each fungus were prepared for the experiment. 

### 3.4. Trichoderma Extracts

Spores isolated from 7 days cultures grown on ZT agar slants (containing (in g/L): glucose, 4; Difco yeast extract, 4; agar, 25; malt extract, 6° Balling (BLG) up to 1 L (1° BLG = 1 g of soluble substances extracted from the grain per 100 mL of malt extract); pH 7.0) were used in the study.

The fungal spores inoculated in 20 mL Sabouraud dextrose broth medium (Difco) were added to 100 mL Erlenmeyer flasks [5]. The spores were cultured on a rotary shaker (160× *g*) for 48 h at 28 °C. Then, 2 mL of preculture was added to the Sabouraud medium. The cultures were incubated on a rotary shaker (160× *g*) at 28 °C. Following 24 h of incubation, the fungal cultures were filtered through a 115 mL filter unit (Thermo Scientific, Waltham, MA, USA), then 10 mL of the supernatant was transferred to a 50 mL Falcon tube. 

The extracts were prepared according to the QuEChERS method described in our previous publication [3]. A 24 h *T. harzianum* culture was used for the study. After filtration, the supernatant was transferred into a 50 mL tube and 10 mL acetonitrile and the mix of salts (2 g MgSO_4_, 0.5 g NaCl, 0.5 g C_6_H_5_Na_3_O_7_ × 2H_2_O, 0.25 g C_6_H_6_Na_2_O_7_ × 1.5H_2_O) were added. The tubes were shaken for 20 min using a rotary laboratory mixer. Subsequently, the tubes were centrifuged at 4000× *g* for 10 min at 4 °C, then the upper phase was transferred to new tubes and evaporated under pressure. Each extract was dissolved in 3 mL of solution, half of which was used for the one plant sample.

### 3.5. Relative Water Content

The relative water content (RWC) was determined on the 7th day of cultivation according to the Manzoni method [6]. The first component of the equation was the fresh plant shoot weight (W). According to the method used by Arndt et al. [45], the shoots were hydrated to full turgidity for 2 h, subsequently weighed, then the second parameter (TW) was obtained. Then, the plant material was put into a laboratory oven at –80 °C for 24 h. When the shoots were completely dried and cooled, the last parameter (dry weight) was assessed (DW). 

The RWC was determined according to the equation:

RWC (%) = [(W − DW)/(TW − DW)] × 100

The experiment was carried out three times and a total of 30 shoots were studied in each sample. 

### 3.6. Chlorophyll Content Determination 

The chlorophyll amount was determined after 7 days of cultivation. For this study, a Chlorophyll Content Meter CCM-300 (Opti-Sciences (Hudson, NY, USA)) was used. The measurement determined the CFR (the emission ratio of fluorescence) at wavelengths of 735 and 700 nm and the chlorophyll content is expressed in mg/m^2^ based on the Gitelson equation. The measurements were carried out 4 times in three independent repetitions. 

### 3.7. Metabolite Determination

Zearalenone was extracted from the filter paper. After 7 days of plant cultivation, the filter paper from Petri dishes was transferred to 50 mL tubes, then 5 mL of water and glass beads were added to each sample. The filter papers were crushed in a laboratory ball mill at a frequency of 30 min^−1^ for 3 min. Then, 10 mL of acetonitrile was added and the tubes were shaken again with the same parameters. The QuEChERS method (see Section 2.4) was performed with the obtained mixtures and the extracts were stored at −20 °C before analysis. 

For zearalenone determination, the LC-MS/MS methods were applied. The extract was fractioned using the Agilent 1200 HPLC system (Agilent, Santa Clara, CA, USA). A Kinetex C18 column (50 mm × 2.1 mm, particle size: 5 μm; Phenomenex, (Torrance, CA, USA); column temperature 40 °C, injection volume 10 µL) was applied for chromatographic separation. The eluents used in the study were water (A) and methanol (B), both containing 5 mM ammonium formate. The solvent was eluted at a constant flow rate of 500 µL min^−1^, starting with 80% of eluent A for 0.25 min, which then decreased to 10% of eluent A and was maintained for 4 min. The initial conditions were restored for a further 2 min. The MS/MS detection was performed using multiple reaction monitoring (MRM) mode during negative ionization. The monitored MRM pairs were m/z 316.9 > 130.5 and 316.9 > 174.1 for zearalenone.

For *Trichoderma* metabolites, the same chromatographic steps were performed. The MS/MS detection was conducted using MRM mode during positive ionization [3].

### 3.8. CAT and SOD Activity 

The enzymes activity study was performed according to Moura et al.’s method [46]. Briefly, 200 mg of fresh plant biomass (with shoots and roots separated) was ground in a mortar with freeze-drying in liquid nitrogen, then 1 mL of extraction buffer (0.05 M sodium phosphate buffer pH 7.8, 1 mM EDTA, 1%PVP) was added to the powdered tissue. The mixture was collected in the 1.5 mL tubes and centrifuged at 10,000× *g* at 4 °C for 10 min. After the centrifugation, the supernatant was transferred into new tubes. The enzymes extracts were kept on ice. 

The activity of catalase (CAT) was determined by measuring H_2_O_2_ degradation at λ240. The superoxide dismutase (SOD) activity was measured by spectrophotometric evaluation of the inhibition of nitrotetrazolium blue (NBT) chloride reduction at λ540 [47]. 

### 3.9. Oxylipin and Hormone Extraction and Determination

Oxylipins were extracted from the roots or shoots according to the method used by Salem et al. with slight modifications [48]. Here, 100 mg of fresh plant tissue was ground in a mortar with freeze-drying in liquid nitrogen. The plant powder was transferred into 2 mL tubes and 1 mL of MTBE/methanol solution (3:1) was added. The tubes were mixed in an orbital laboratory shaker for 30 min and centrifuged at 10,000× *g* at 4 °C for 10 min. The supernatant was partitioned to two new tubes, one of which was left at room temperature until dryness and transferred to −20 °C for further analysis. Before the oxylipin determination, the samples were defrosted and dissolved in methanol.

In the second tube with a half portion of the supernatant, 0.5 mL of 0.1% water HCl solution was added, then the tubes were shaken for 30 min in an orbital shaker for hormones analysis followed by centrifugation. The upper phase was dried at room temperature for 24 h and stored at −20 °C for further analysis. Before the hormone determination the samples were defrosted and dissolved in methanol.

Oxylipins were measured according to the method described in our previous work [3] using an Agilent 1200 HPLC system and a 4500 Q-TRAP mass spectrometer (Sciex, Foster City, CA, USA) with an ESI source. 

### 3.10. Protein Extraction

Protein extraction from the plant tissue was based on the modified method used by Zhang et al. [49]. The previously prepared plant material was freeze-dried in liquid nitrogen and ground in a mortar until the tissue powder was obtained. A total of 0.25 g of the ground material was transferred into 2 mL low-binding Eppendorf tubes. Next, 1 mL of ice-cold 50% TCA/acetone with 1% ß-mercaptoethanole was added, vortexed for 3 min, chilled on ice for 5 min, then centrifuged in a pre-cooled rotor at 13,500× *g* at 4 °C for 5 min. Then, the supernatant was removed and the last step was repeated until the samples were completely discolored. In further proceedings, the pellets were air-dried in the tubes at room temperature to remove the acetone residue. Next, 700 μL phenol with 0.5% DTT was added and the samples were incubated in an ultrasonic laboratory bath for 10 min, with the temperature kept below 30 °C. The centrifugation was performed according to the same parameters and the supernatant was placed in new tubes. The last step was repeated 3 times and the collected supernatant was transferred into new 2 mL tubes in an equal amount of 300 μL. For the precipitation step, methanol with 0.1% ammonium acetate was added into a volume of 5 ml, then an equivalent of the supernatant was supplemented and the tubes were incubated at 20 °C overnight. After the precipitation, the samples were centrifuged in a pre-cooled rotor at 15,000× *g* at 4 °C for 20 min and the supernatant was removed. 

For the purification step, the precipitates were collected in one tube within one sample and filled with 500 μL of guanidine hydrochloride (8M), then vortexed until the pellet dissolved. Next, tributyl phosphate (final concentration 5 mM) and 2-Vinyl-pyridin (final concentration 100 mM) were added. The samples were vortexed for 30 min in the dark and centrifuged as described earlier. The supernatant was transferred into two new tubes in equal amounts and 5 volumes of precooled acetone/methanol (1:1) were added. The precipitation was performed at −20 °C for 20 min and centrifugation at 13,500× *g* was carried out at 4 °C for 10 min. After the supernatant was removed, the pellets were collected in one tube within one sample, rinsed with methanol, vortexed for 3 min, and centrifuged on the same parameters as in the last step. This stage was performed three times. After the last centrifugation phase, the samples were dried in thermoblock and diluted in rehydration buffers (7 M urea, 2 M thiourea, 4% CHAPS, 0.01 M DTT). The samples prepared in this way were stored at −70 °C until analysis.

### 3.11. 2-D SDS PAGE 

Two-dimensional sodium dodecyl sulfate–polyacrylamide gel electrophoresis (2D SDS PAGE) was conducted to separate the spots. Next, 300 μg of protein in 200 μL rehydration buffer was mixed with 7.5 μL of 40% ampholytes (pH range ~3.5–9.5) and 2 μL of bromophenol blue (1%). The full volume mix was loaded onto 11 cm IPG strips of pH 3–10 NL (non-linear) (cat no.163-2016, Bio-Rad, Germany) for overnight rehydration. Isoelectric focusing was performed using a Protean i12 device (Bio-Rad, Germany). The assay was conducted according to the following parameters: 50 V for 4 h, linear gradient to 8000 V for 5 h, then held for 76,000 Vh. In the next step, the strips were equilibrated according to the Bio-Rad gel preparation protocol and the strips were placed on several polyacrylamide gels. As a gel calibrator, a 6500–200,000-Da molecular mass marker (Sigma-Aldrich) was used. 

Same Spots software (the United Kingdom) was used to identify differences. The spots that differed in intensity were excised from the gels, digested, and used for further analyses.

### 3.12. Protein Identification

After excising the spots from the 2-DE gels manually, they were transferred to Eppendorf tubes (2.0 mL) for digestion as described by Szewczyk et al. [50]. Spectra were obtained using matrix-assisted laser desorption ionization–time of flight (MALDI-TOF)/TOF (Sciex 5800 TOF/TOF system, Foster City, CA, USA) as described by Bernat et al. [51].

Protein Pilot v4.5 (Sciex) with the Mascot software v2.4 (Matrix Science, London, UK) was used for protein identification. The MS data were analyzed using the NCBI database with a taxonomy filter for *Triticum aestivum* (total number of sequences = 684,919).

To obtain the information on the functions of hypothetical proteins, the BLASTp algorithm in the nonredundant BLAST protein database was used. All searches were evaluated based on the significant scores obtained from MASCOT. The protein score was set to >95% and a significance threshold of *p* < 0.05 was used.

### 3.13. Statistical Analysis

In the germination study, growth condition parameters were measured and statistically analyzed for 20 seedlings, whereas all metabolomic experiments were carried out in three replicates. The standard error was determined and marked in the figures as an error bar. For more precise statistical analysis, the STATISTICA c.13.3 software was used. The study was performed using analysis of variance with Tukey’s post hoc test. The data were considered as significant at *p* < 0.05. The results for all subcolumn analyses are attached as Supplementary Materials.

For the proteomic study, the proteins with scores greater than 68 (5% confidence threshold) were included as statistically significant. 

## 4. Conclusions

This work was focused on the examination of how *T. harzianum* and its bioactive metabolites might influence germinated wheat defense against chemical stress (2,4-D) and biological infection (*F. culmorum*). The results indicated that the use of *Trichoderma* fungus or its extracellular metabolites could be an effective stimulant for plant development, as well as for the improvement of overall grain germination. The use of a living organism has proven to be a more effective method for minimizing oxidative stress in wheat caused by the interaction with *F. culmorum*. The obtained proteomics results confirmed the changes in the oxylipin profiles and the activity levels of CAT and SOD in the ROS scavenging mechanism. In the future, we plan to check the possibility of using *T. harzianum* for longer cultivation periods in order to trace further changes and to provide a more stable method for using metabolites.

## Figures and Tables

**Table 1:** Changes in average protein spot volumes, after SDS-Page electrophoresis and trypsin digestion analyzed by MALDI TOF/TOF identified by Mascot from root and shoot material of wheat, analyzed after 7 days of cultivation (T. h.—*T. harzianum* inoculated wheat seeds, F. c.—*F. culmorum* inoculated wheat seeds, ex. T. h.—*T. harzianum* extracellular metabolites treated wheat seeds).

Spot ID	Accession Number *	Theo-Retical MW [Da]	Score **	Protein	Function	Examinated Avarege ROOT Sample Volumes ***												*p* Value ****
						Control	2,4D	T.h.	T.h.+ 2,4D	F.c.	F.c.+ 2,4D	T.h.+F.c.	T.h.+F.c.+ 2,4D	ex.T.h.	ex.T.h.+2,4D	ex.T.h.+F.c.	ex.T.h.+F.c.+2,4D	
1	AAB67990.1	20,310	106	Cu/Zn superoxide dismutase	Oxidoreductase/responce to oxidative stress	5.86 × 10^4^	2.53 × 10^4^	1.63 × 10^4^	7.07 × 10^4^	6.90 × 10^5^	1.56 × 10^5^	3.95 × 10^5^	4.88 × 10^5^	1.80 × 10^4^	1.88 × 10^4^	2.29 × 10^5^	2.83 × 10^5^	0.01
2	KAF7042201.1	26,606	493	Ascorbate peroxidases and cytochrome C peroxidases		1.14 × 10^4^	6.77 × 10^3^	9.58 × 10^3^	6.00 × 10^4^	6.31 × 10^4^	1.25 × 10^5^	1.09 × 10^5^	1.14 × 10^5^	1.83 × 10^4^	1.79 × 10^5^	3.87 × 10^4^	3.31 × 10^4^	0.03
3	KAF7054315.1	27,470	505	catalase/peroksidase HPI		4.14 × 10^4^	9.32 × 10^4^	1.37 × 10^4^	6.32 × 10^4^	7.14 × 10^4^	1.72 × 10^5^	1.12 × 10^5^	1.35 × 10^5^	3.93 × 10^4^	8.35 × 10^4^	6.34 × 10^4^	5.24 × 10^4^	0.02
4	ACF70712.1	32,429	142	root peroxidase		1.73 × 10^4^	1.41 × 10^4^	5.06 × 10^4^	1.55 × 10^4^	8.83 × 10^3^	4.62 × 10^3^	6.93 × 10^3^	3.14 × 10^3^	6.54 × 10^4^	4.41 × 10^4^	5.13 × 10^3^	3.93 × 10^3^	0.007
5	KAF6989700.1	16,607	375	nucleoside diphosphate kinase		1.38 × 10^5^	4.98 × 10^4^	2.20 × 10^5^	2.08 × 10^5^	2.10 × 10^4^	4.14 × 10^4^	8.01 × 10^4^	7.09 × 10^4^	4.63 × 10^4^	3.51 × 10^4^	2.67 × 10^4^	2.06 × 10^4^	0.02
6	AAF64241.1	25,277	156	cytosolic glyceraldehyde-3-phosphate dehydrogenase GAPDH		8.58 × 10^4^	1.51 × 10^5^	7.68 × 10^4^	8.64 × 10^4^	9.05 × 10^4^	5.45 × 10^4^	8.91 × 10^4^	6.61 × 10^4^	6.03 × 10^4^	1.38 × 10^5^	1.17 × 10^5^	8.33 × 10^4^	0.01
7	CAI47635.1	36,482	107	peroxidase precursor		1.18 × 10^4^	1.24 × 10^4^	1.62 × 10^4^	3.65 × 10^4^	6.58 × 10^4^	4.20 × 10^4^	5.65 × 10^4^	9.05 × 10^4^	2.13 × 10^4^	1.63 × 10^4^	3.23 × 10^4^	3.96 × 10^4^	0.03
8	KAF6996030.1	33,096	114	NmrA-like family		5.02 × 10^4^	2.44 × 10^4^	6.37 × 10^4^	3.30 × 10^4^	9.64 × 10^4^	1.13 × 10^5^	1.04 × 10^5^	1.02 × 10^5^	1.62 × 10^4^	1.38 × 10^4^	9.83 × 10^4^	7.66 × 10^4^	0.03
9	AFC87832.1	40,578	80	12-oxo-phytodienoic acid reductase	Oxylipin biosynthesis	4.75 × 10^4^	1.86 × 10^5^	3.27 × 10^4^	1.89 × 10^5^	1.78 × 10^5^	1.15 × 10^5^	1.24 × 10^5^	1.42 × 10^5^	2.15 × 10^5^	1.79 × 10^5^	8.76 × 10^4^	1.36 × 10^5^	0.03
10	AAC23502.1	56,398	97	vacuolar invertase, partial	Carbohydrate metabolism	1.06 × 10^4^	7.49 × 10^3^	1.64 × 10^4^	1.25 × 10^4^	5.39 × 10^3^	8.02 × 10^3^	1.30 × 10^4^	8.08 × 10^3^	3.23 × 10^3^	4.99 × 10^3^	6.66 × 10^3^	5.64 × 10^3^	0.02
11	AGN71004.1	33,254	130	xylanase inhibitor protein precursor		1.18 × 10^4^	2.05 × 10^4^	7.92 × 10^3^	4.49 × 10^3^	1.15 × 10^4^	5.50 × 10^3^	5.18 × 103	1.27 × 10^4^	6.75 × 10^3^	1.84 × 10^3^	1.63 × 10^4^	9.71 × 10^3^	0.03
12	ATY36097.1.	47,317	408	alpha-amylase		1.02 × 10^5^	1.34 × 10^5^	3.14 × 10^5^	2.23 × 10^5^	2.55 × 10^5^	1.16 × 10^5^	1.79 × 10^5^	1.49 × 10^5^	4.46 × 10^5^	2.70 × 10^5^	2.78 × 10^5^	1.88 × 10^5^	0.04
13	KAF6992161.1	24,332	525	malate dehydrogenase		8.16 × 10^4^	1.75 × 10^5^	1.87 × 10^5^	1.98 × 10^5^	1.63 × 10^5^	1.30 × 10^5^	1.46 × 10^5^	1.10 × 10^5^	9.40 × 10^4^	1.19 × 10^5^	2.36 × 10^5^	1.84 × 10^5^	0.03
14	AAX83262.1	28,242	83	class II chitinase		9.93 × 10^4^	4.25 × 10^4^	1.19 × 10^5^	4.78 × 10^4^	3.37 × 10^5^	2.89 × 10^5^	5.19 × 10^5^	2.40 × 10^5^	2.14 × 105	1.20 × 10^5^	3.84 × 10^4^	4.33 × 10^4^	0.03
15	ABY85789.1	43,153	366	S-adenosylmethionine synthetase		3.03 × 10^5^	1.89 × 10^5^	1.94 × 10^5^	1.13 × 10^5^	1.62 × 10^5^	1.43 × 10^5^	2.00 × 10^5^	8.32 × 10^4^	1.48 × 105	1.83 × 10^5^	1.96 × 10^5^	1.74 × 10^5^	0.02
16	CAC94001.1	24,984	324	glutathione transferase	Glutathione metabolic process	6.70 × 10^3^	3.71 × 10^4^	1.99 × 10^4^	1.74 × 10^4^	7.68 × 10^3^	2.32 × 10^4^	1.50 × 10^4^	2.67 × 10^4^	2.78 × 10^4^	1.35 × 10^4^	5.18 × 10^3^	7.73 × 10^3^	0.03
17	AAL71854.1	23,343	277	dehydroascorbate reductase		1.28 × 10^4^	1.45 × 10^4^	3.10 × 10^4^	1.16 × 10^4^	9.22 × 10^3^	8.29 × 10^3^	8.98 × 10^3^	1.50 × 10^4^	1.02 × 10^4^	7.24 × 10^3^	3.14 × 10^3^	4.60 × 10^3^	0.03
18	CAC14917.1	26,786	197	triosephosphat-isomerase	Glycolytic process	1.41 × 10^5^	1.02 × 10^5^	6.86 × 10^4^	1.31 × 10^5^	3.16 × 105	3.49 × 10^5^	4.62 × 10^5^	3.44 × 10^5^	4.21 × 10^4^	1.13 × 10^5^	2.98 × 10^4^	2.31 × 10^4^	0.03
19	AVL25146.1	38,772	119	fructose-1.6 -bisphosphate aldolase		1.37 × 10^4^	2.37 × 10^4^	5.92 × 10^4^	6.02 × 10^4^	1.23 × 10^4^	1.55 × 10^4^	2.65 × 10^4^	6.65 × 10^4^	1.64 × 10^4^	7946.144	4.29 × 10^4^	7.13 × 10^4^	0.03
20	AAP80633.1	29,558	265	phosphoglycerate mutase, partial		2.59 × 10^4^	3.16 × 10^4^	5.61 × 10^4^	2.06 × 10^4^	3.32 × 10^4^	4.40 × 10^4^	4.22 × 10^4^	3.22 × 10^4^	4.21 × 10^4^	4.81 × 10^4^	7.30 × 10^4^	6.48 × 10^4^	0.03
21	ALE18234.1	25,277	330	glyceraldehyde-3-phosphate dehydrogenase GAPDH		1.83 × 10^6^	1.33 × 10^6^	1.73 × 10^6^	1.66 × 10^6^	1.30 × 10^6^	1.10 × 10^6^	7.96 × 10^5^	1.23 × 10^6^	8.86 × 10^5^	1.07 × 10^6^	1.08 × 10^6^	9.68 × 10^5^	0.02
22	AWS00780.1	41,701	151	actin	ATP-binding	3.84 × 10^4^	3.14 × 10^4^	3.83 × 10^4^	2.00 × 10^4^	3.24 × 10^4^	3.52 × 10^4^	4.30 × 10^4^	3.14 × 10^4^	1.98 × 10^4^	2.52 × 10^4^	4.93 × 10^4^	4.41 × 10^4^	0.04
23	AGN94842.1	73,171	597	ER molecular chaperone	ATPase activity	3.72 × 10^4^	8.52 × 10^4^	4.34 × 10^4^	3.70 × 10^4^	7.83 × 10^4^	3.89 × 10^4^	2.42 × 10^4^	2.60 × 10^4^	2.58 × 10^4^	8.12 × 10^4^	2.88 × 10^4^	4.69 × 10^4^	0.02
24	CAA52636.1	59,212	1090	ATP synthase beta subunit		5.10 × 10^5^	6.77 × 10^5^	4.63 × 10^5^	1.12 × 105	1.53 × 10^5^	1.62 × 10^5^	1.72 × 10^5^	1.56 × 10^5^	3.25 × 10^5^	2.05 × 10^5^	1.46 × 10^5^	1.12 × 10^5^	0.001
25	ADK35122.1	14,182	203	profilin	Actin binding	8.30 × 10^4^	3.16 × 10^4^	6.87 × 10^4^	5.20 × 10^4^	2.54 × 10^4^	3.99 × 10^4^	3.72 × 10^4^	2.60 × 10^4^	1.03 × 10^4^	2.28 × 10^4^	1.08 × 10^4^	1.83 × 10^4^	0.03
26	AIZ95472.1	16,047	141	actin-depolymerizing factor 3		3.63 × 10^3^	2.12 × 10^3^	1.97 × 10^3^	1.40 × 10^3^	2.96 × 10^3^	5.60 × 10^3^	2.37 × 10^3^	1.56 × 10^3^	1.43 × 10^3^	1.45 × 10^3^	1.94 × 10^3^	1.60 × 10^3^	0.03
27	AAA75104.1	16,245	71	single-strained nucleic acid binding protein	RNA-binding	3.93 × 10^4^	2.99 × 10^4^	2.85 × 10^4^	9.71 × 10^3^	2.54 × 10^4^	3.99 × 10^4^	3.73 × 10^4^	2.60 × 10^4^	1.07 × 10^4^	1.91 × 10^4^	1.08 × 10^4^	1.83 × 10^4^	0.03
28	AAA80609.1	19,651	158	adenine phosphoribosyltransferase form 1	Adenine salvage	6.02 × 10^3^	2.05 × 10^3^	9.80 × 10^3^	1.57 × 10^3^	8.17 × 10^3^	6.19 × 10^3^	8.77 × 10^3^	2.48 × 10^3^	8.92 × 10^3^	1.49 × 10^3^	1.17 × 10^4^	5.37 × 10^3^	0.01
29	CAA46811.1	38,331	123	cathepsin	Regulation of catalytic activity	3.25 × 10^4^	3.10 × 10^4^	6.11 × 10^4^	2.68 × 10^4^	4.28 × 10^4^	2.70 × 10^4^	5.19 × 10^4^	2.10 × 10^4^	2.72 × 10^4^	5.22 × 10^4^	3.04 × 10^4^	3.35 × 10^4^	0.03
30	ABB80135.1	26,160	179	vacuolar proton ATPase factor 3	Ion transport	4.46 × 10^3^	3.20 × 10^3^	3.24 × 10^3^	7.57 × 10^3^	1.73 × 10^4^	1.15 × 10^4^	2.49 × 10^4^	2.19 × 10^4^	1.06 × 10^4^	3.36 × 10^3^	1.33 × 10^4^	6.70 × 10^3^	0.02
31	KAF7056598.1	82,549	193	5-methyltetrahydropteroyltriglutamate homocysteine methyltransferase	Amino-acid biosynthesis	1.43 × 10^3^	1.88 × 10^4^	4.71 × 10^3^	3.30 × 10^3^	2.17 × 10^3^	3.16 × 10^3^	4.54 × 10^3^	7.28 × 10^3^	4.91 × 10^3^	4.97 × 10^3^	2.34 × 10^3^	4.45 × 10^3^	0.01
32	AAZ95171.1	17,352	162	eukaryotic translation initiation factor	Protein biosynthesis	6.13 × 10^4^	9.50 × 10^4^	1.93 × 10^4^	5.32 × 10^4^	3.92 × 10^4^	4.93 × 10^4^	8.33 × 10^4^	7.09 × 10^4^	1.61 × 10^4^	2.23 × 10^4^	4.35 × 10^4^	3.33 × 10^4^	0.03
33	AAS17067.1	18,367	228	cyclophilin A	Protein folding	3.27 × 10^5^	5.52 × 10^4^	5.13 × 10^4^	1.24 × 10^5^	5.43 × 10^4^	6.52 × 10^4^	9.64 × 10^3^	4.07 × 10^4^	5.94 × 10^4^	9.39 × 10^4^	5.12 × 10^4^	3.71 × 10^4^	0.04
34	ABQ51156.1	24,404	184	Triticin, partial	Storage protein	1.65 × 10^3^	8.33 × 102	1.64 × 10^3^	2.07 × 10^3^	1.51 × 10^4^	1.33 × 10^3^	2.94 × 10^3^	2.55 × 10^3^	1.27 × 10^3^	7.88 × 102	3.18 × 10^3^	4.60 × 10^3^	0.03
35	ADQ85915.1	15,293	125	abscisic stress-ripening protein	Stress response	4.02 × 10^3^	6.95 × 10^3^	2.21 × 10^4^	8.49 × 10^3^	2.71 × 10^3^	5.48 × 10^3^	1.17 × 10^4^	3.43 × 10^3^	5.58 × 10^3^	2.21 × 10^3^	3.07 × 10^3^	4.06 × 10^3^	0.02
36	ADN05856.1	65,057	78	HOP		6.88 × 10^3^	9.09 × 10^3^	2.01 × 10^4^	7.43 × 10^3^	6.42 × 10^3^	3.40 × 10^3^	6.16 × 10^3^	1.14 × 10^4^	1.25 × 10^4^	1.46 × 10^4^	1.93 × 10^3^	3.86 × 10^3^	0.03
37	KAF7107406.1	25,857	161	co-chaperonin		1.01 × 10^4^	6.21 × 10^3^	1.81 × 10^3^	5.95 × 10^3^	1.71 × 10^3^	2.50 × 10^3^	2.14 × 10^3^	2.79 × 10^3^	2.53 × 10^3^	4.21 × 10^3^	2.08 × 10^3^	1.86 × 10^3^	0.04
38	ACQ41884.1	23,369	212	germin-like protein		1.68 × 10^5^	2.38 × 10^5^	5.22 × 10^5^	1.71 × 10^3^	6.47 × 10^5^	6.34 × 10^5^	2.70 × 10^5^	3.87 × 10^5^	1.43 × 10^5^	3.70 × 10^5^	4.99 × 10^5^	3.51 × 10^5^	0.01
39	KAF7063064	32,946	78	allergenic/antifungal thaumatin-like proteins	Defence response	1.07 × 10^5^	1.51 × 10^5^	6.64 × 10^4^	3.74 × 10^4^	1.14 × 10^5^	1.55 × 10^5^	1.30 × 10^5^	1.25 × 10^5^	2.03 × 10^5^	1.21 × 10^5^	5.46 × 10^4^	6.45 × 10^4^	0.03
40	ABX89061.1	17,023	147	pathogenesis-related protein		1.65 × 10^3^	9.42 × 10^2^	2.18 × 10^3^	1.07 × 10^3^	3.66 × 10^3^	2.86 × 10^3^	1.43 × 10^3^	9.59 × 10^2^	6.73 × 10^2^	2.95 × 10^3^	7.48 × 10^2^	7.57 × 10^2^	0.02
41	AFC89429.1	42,969	77	serpin-N3.2	Endopeptidase inhibitor activity	5.11 × 10^3^	5.73 × 10^3^	1.98 × 10^4^	4.96 × 10^3^	1.24 × 10^4^	2.89 × 10^4^	1.15 × 10^4^	4.72 × 10^3^	2.99 × 10^3^	5.77 × 10^3^	7.34 × 10^3^	3.79 × 10^3^	0.03
101	AFF19563.1	20,310	138	superoxide dismuase	Oxidoreductase	2.55 × 10^4^	6.75 × 10^3^	7.35 × 10^3^	7.31 × 10^3^	3.11 × 10^4^	4.78 × 10^4^	2.18 × 10^4^	2.68 × 10^4^	4.71 × 10^3^	8.16 × 10^3^	4.42 × 10^4^	4.06 × 10^4^	0.01
	102	CAI47635.1	36,482	146	peroxidase precursor	6.18 × 10^4^	9.25 × 10^4^	7.11 × 10^4^	6.63 × 10^4^	4.40 × 10^4^	2.24 × 10^4^	4.25 × 10^4^	4.06 × 10^4^	1.55 × 10^5^	9.70 × 10^4^	3.76 × 10^4^	6.98 × 10^4^	0.04
	103	ACO90196.1	26,606	151	ascorbate peroxidase	2.60 × 10^4^	1.29 × 10^4^	5.55 × 10^3^	5.47 × 10^4^	3.09 × 10^4^	5.13 × 10^4^	4.23 × 10^4^	1.35 × 10^4^	1.32 × 10^4^	4.29 × 10^4^	1.13 × 10^4^	4.66 × 10^4^	0.001
	104	CDX58685.1	41,427	155	RUBISCO activase beta	4.04 × 10^4^	1.03 × 10^5^	1.61 × 10^5^	7.41 × 10^4^	7.64 × 10^4^	4.87 × 10^4^	6.39 × 10^4^	5.47 × 10^4^	1.13 × 10^5^	1.34 × 10^5^	9.84 × 10^4^	6.55 × 10^4^	0.03
105	AAM88439.1	23,711	76	putative Rieske Fe-S precursor protein	Photosynthetic electron transport chain	2.41 × 10^4^	8.57 × 10^3^	2.52 × 10^4^	2.84 × 10^4^	2.46 × 10^4^	6.06 × 10^4^	5.65 × 10^4^	5.00 × 10^4^	1.18 × 10^4^	1.29 × 10^4^	4.71 × 10^4^	3.58 × 10^4^	0.01
106	ARQ82872.1	27,184	483	oxygen evolving enhancer protein		1.21 × 10^6^	6.33 × 10^5^	1.28 × 10^6^	7.78 × 10^5^	1.42 × 10^5^	1.65 × 10^5^	1.25 × 10^5^	1.15 × 10^5^	1.25 × 10^6^	1.16 × 10^6^	6.60 × 10^4^	1.05 × 10^5^	0.01
107	BAA35176.1	52,817	407	ribulose-1.5 -bisphosphate arboxylase/oxygenase small subunit		3.57 × 10^5^	3.24 × 10^5^	5.41 × 10^5^	5.32 × 10^5^	4.02 × 10^4^	4.80 × 10^4^	4.22 × 10^4^	6.83 × 10^4^	2.28 × 10^5^	1.82 × 10^5^	9.59 × 10^4^	3.96 × 10^4^	0.04
108	CDX48684.1	44,554	312	RUBISCO activase alpha		3.12 × 10^4^	6.98 × 10^4^	4.68 × 10^4^	2.32 × 10^4^	4.20 × 10^4^	5.16 × 10^4^	4.80 × 10^4^	3.97 × 10^4^	4.88 × 10^4^	5.88 × 10^4^	7.34 × 10^4^	8.21 × 10^4^	0.03
109	KAF7080451.1	17,287	92	glycine cleavage system protein GcvH		1.66 × 10^5^	3.12 × 10^4^	1.98 × 10^4^	3.45 × 10^4^	4.79 × 10^4^	1.79 × 10^4^	2.31 × 10^4^	3.56 × 10^4^	3.25 × 10^4^	5.19 × 10^4^	3.14 × 10^4^	5.14 × 10^4^	0.04
110	KAF7061990.1	15,592	357	photosystem I reaction center subunit IV		1.69 × 10^5^	9.70 × 10^4^	1.22 × 10^5^	1.43 × 10^5^	6.19 × 10^3^	6.86 × 10^3^	8.34 × 10^3^	9.16 × 10^3^	2.95 × 10^4^	4.48 × 10^4^	1.62 × 10^4^	9.58 × 10^4^	0.03
111	ABB80135.1	26,160	198	vacuolar proton ATPase subunit	Ion transport	3.75 × 10^4^	3.90 × 10^4^	3.45 × 10^4^	3.76 × 10^4^	2.16 × 10^4^	1.26 × 10^4^	3.25 × 10^4^	9.64 × 10^3^	3.51 × 10^4^	2.76 × 10^4^	1.09 × 10^4^	2.22 × 10^4^	0.001
112	ACV89491.1	23,343	300	dehydroascorbate reductase	glutathione metabolism	3.42 × 10^4^	4.40 × 10^4^	3.84 × 10^4^	1.78 × 10^4^	1.80 × 10^5^	2.65 × 10^5^	1.52 × 10^5^	1.98 × 10^5^	1.33 × 10^4^	2.21 × 10^4^	3.78 × 10^5^	3.26 × 10^5^	0.03
113	CAC94001.1	24,984	132	glutathione transferase		5.22 × 10^4^	1.50 × 10^4^	2.04 × 10^4^	1.15 × 10^4^	1.85 × 10^4^	1.14 × 10^4^	2.05 × 10^4^	2.16 × 10^4^	2.18 × 10^4^	1.15 × 10^4^	1.24 × 10^4^	1.05 × 10^4^	0.04
114	AAA75104.1	16,245	486	single-stranded nucleic acid binding protein	RNA binding	9.93 × 10^4^	1.77 × 10^4^	1.33 × 10^4^	1.94 × 10^4^	4.71 × 10^5^	3.37 × 10^5^	4.22 × 10^5^	3.33 × 10^5^	1.60 × 10^4^	1.95 × 10^4^	3.13 × 10^5^	3.49 × 10^5^	0.02
115	AKQ09032.1	33,413	110	chitinase	carbohydrate metabolism	1.43 × 10^4^	1.63 × 10^4^	1.33 × 10^4^	9.16 × 10^3^	5.25 × 10^4^	4.28 × 10^4^	6.19 × 10^4^	7.97 × 10^4^	7.56 × 10^4^	2.09 × 10^4^	7.30 × 10^4^	1.02 × 10^5^	0.03
116	AAP70009.1	24,332	129	cytosolic malate dehydrogenase		4.41 × 10^4^	8.14 × 10^4^	8.81 × 10^4^	7.54 × 10^4^	7.72 × 10^3^	1.34 × 10^4^	8.40 × 10^3^	1.87 × 10^4^	5.47 × 10^4^	1.24 × 10^5^	1.95 × 10^4^	2.69 × 10^4^	0.04
117	ALE18234.1	36,586	394	glyceraldehyde-3-phosphate dehydrogenase	Glucose metabolic process	2.95 × 10^5^	2.40 × 10^5^	5.63 × 10^5^	3.66 × 10^5^	1.39 × 10^5^	1.40 × 10^5^	1.37 × 10^5^	1.99 × 10^5^	4.85 × 10^5^	2.64 × 10^5^	1.59 × 10^5^	1.41 × 10^5^	0.04
118	AVL25141.1	41,609	104	fructose-1.6 -bisphosphate aldolase		1.08 × 10^4^	2.51 × 10^4^	1.18 × 10^4^	6183	1.78 × 10^4^	2.01 × 10^4^	2.32 × 10^4^	1.62 × 10^4^	2.93 × 10^4^	1.72 × 10^4^	2.18 × 10^4^	1.27 × 10^4^	0.03
119	AEH16638.1	46,673	180	glutamine synthase	Nitrogen metabolism	1.09 × 10^5^	1.00 × 10^5^	2.23 × 10^5^	9.28 × 10^4^	4.96 × 10^4^	7.22 × 10^4^	3.97 × 10^4^	7.90 × 10^4^	8.23 × 10^4^	1.14 × 10^5^	1.05 × 10^5^	8.99 × 10^4^	0.04
120	AAP44537.1	25,875	301	cyclophilin-like protein	Protein folding	1.36 × 10^4^	7.53 × 10^3^	4.46 × 10^3^	2.14 × 10^3^	4.03 × 10^3^	1.65 × 10^3^	4.90 × 10^3^	4.02 × 10^3^	4.29 × 10^3^	5.57 × 10^3^	1.63 × 10^3^	5.73 × 10^3^	0.02
121	AAS17067.1	18,367	165	cyclophilin A		5.83 × 10^4^	1.28 × 10^5^	1.51 × 10^5^	1.41 × 10^5^	4.30 × 10^4^	3.00 × 10^4^	3.50 × 10^4^	4.99 × 10^4^	2.10 × 10^4^	2.91 × 10^4^	3.22 × 10^4^	8.88 × 10^4^	0.03
122	AIZ95472.1	16,047	148	actin depolymerizing factor 3	Actin binding	1.21 × 10^6^	6.33 × 10^5^	1.28 × 10^6^	7.78 × 10^5^	7.49 × 10^5^	7.00 × 10^5^	6.16 × 10^5^	5.84 × 10^5^	1.25 × 10^6^	1.16 × 10^6^	6.88 × 10^5^	6.92 × 10^5^	0.01
123	CAA52636.1	59,212	545	ATP synthase beta subunit	ATPase activity	3.50 × 10^4^	7.62 × 10^4^	8.60 × 10^4^	1.25 × 10^5^	1.28 × 10^5^	1.23 × 10^5^	1.20 × 10^5^	1.37 × 10^5^	5.98 × 10^4^	1.04 × 10^5^	1.51 × 10^5^	8.25 × 10^4^	0.02
124	AND74687.1	47,066	177	chloroplast ribulose bisphosphate carboxylase/oxygenase activase		2.48 × 10^4^	1.24 × 10^4^	2.47 × 10^4^	4.44 × 10^4^	5.40 × 10^4^	7.47 × 10^4^	9.12 × 10^4^	9.74 × 10^4^	8.08 × 10^4^	7.09 × 10^4^	9.38 × 10^4^	9.14 × 10^4^	0.03
125	AGN94842.1	73,141	260	ER molecular charpeone		1.77 × 10^4^	5.49 × 10^4^	2.54 × 10^4^	4.31 × 10^4^	4.02 × 10^4^	4.80 × 10^4^	4.22 × 10^4^	6.83 × 10^4^	2.20 × 10^4^	1.45 × 10^4^	9.59 × 10^4^	3.96 × 10^4^	0.02
126	ABY85789.1	43,153	118	S-adenosylmethionine synthetase	ATP binding	2.87 × 10^4^	6.98 × 10^4^	3.62 × 10^4^	8.69 × 10^4^	1.48 × 10^4^	3.34 × 10^4^	2.08 × 10^4^	2.16 × 10^4^	1.05 × 10^5^	5.97 × 10^4^	5.56 × 10^4^	2.48 × 10^4^	0.04
127	KAF6989700.1	16,607	290	nucleoside diphosphate kinase		1.06 × 10^5^	1.39 × 10^5^	1.29 × 10^5^	1.72 × 10^5^	1.52 × 10^5^	1.19 × 10^5^	1.37 × 10^5^	1.02 × 10^5^	1.30 × 10^5^	4.81 × 10^4^	8.55 × 10^4^	1.16 × 10^5^	0.03
128	CAC85479.1	21,801	155	adenosine diphosphate glucose pyrophosphatase	Biosynthesis of starch	6.45 × 10^4^	5.40 × 10^4^	6.04 × 10^4^	9.81 × 10^4^	2.46 × 10^4^	6.06 × 10^4^	5.65 × 10^4^	5.00 × 10^4^	4.20 × 10^4^	3.75 × 10^4^	4.71 × 10^4^	3.58 × 10^4^	0.04

* Accession number—is a unique identifier given to a protein sequence record, identified by Mascot; ** Score—cumulative scores for individual peptides for all peptides matching a given protein; *** Spot volume—the sum of the pixel intensities within the spot area; **** *p* value between the highest and lowest normalized spot volumes.

## Data Availability

The data presented in this study are available on request from the corresponding author.

## Supplementary Materials

The following are available online at https://www.mdpi.com/article/10.3390/ijms222313058/s1.
