# Supplementary material for: Potential of Trichoderma harzianum and Its Metabolites to Protect Wheat Seedlings against Fusarium culmorum and 2,4-D
Source: Int J Mol Sci. 2021 Dec 2;22(23):13058. doi: 10.3390/ijms222313058 (PMC8657962; doi:10.3390/ijms222313058)
Supplement: Supplementary file 1 [file ijms-22-13058-s001.zip › ijms-1405548-supplementary.pdf]

Supplementary material

Table S1. Comparison of ANOVA Tukey post-hoc results for shoot and root lengths, with treatment as a factor, (T.h. – *Trichoderma harzianum,* F.c.- *Fusarium culmorum,* ex. T.h – *T. harzianum* extracellular metabolites treated wheat seeds), p significance was shown: * - p ≤ 0.001, ns – not significant.

ANOVA result for shoot length

	Control	2,4D	T.h.	T.h.+2,4D	F.c.	F.c.+2,4D	T.h.+F.c.	T.h.+F.c.+2,4D	ex.T.h.	ex.T.h.+2,4D	ex.T.h.+F.c.	ex.T.h.+F.c.+2,4D
Control		*	*	*	*	*	ns	ns	*	*	*	*
2,4D	*		*	ns	ns	ns	*	*	*	ns	ns	ns
T.h.	*	*		*	*	*	ns	*	ns	*	*	*
T.h.+2,4D	*	ns	*		ns	ns	*	ns	*	ns	ns	ns
F.c.	*	ns	*	ns		ns	*	ns	*	ns	ns	ns
F.c.+2,4D	*	ns	*	ns	ns		*	*	*	ns	ns	ns
T.h.+F.c.	ns	*	ns	*	*	*		*	ns	*	*	*
T.h.+F.c.+2,4D	ns	*	*	ns	ns	*	*		*	ns	*	ns
ex.T.h.	*	*	ns	*	*	*	ns	*		*	*	*
ex.T.h.+2,4D	*	ns	*	ns	ns	ns	*	ns	*		ns	ns
ex.T.h.+F.c.	*	ns	*	ns	ns	ns	*	*	*	ns		ns
ex.T.h.+F.c.+2,4D	*	ns	*	ns	ns	ns	*	ns	*	ns	ns	

ANOVA result for root length

	2,4D	Control	T.h.	T.h.+2,4D	F.c.	F.c.+2,4D	T.h.+F.c.	T.h.+F.c.+2,4D	ex.T.h.	ex.T.h.+2,4D	ex.T.h.+F.c.	ex.T.h.+F.c.+2,4D
2,4D		*	*	ns	*	ns	*	ns	*	ns	*	ns
Control	*		*	*	*	*	ns	*	ns	*	*	*
T.h.	*	*		*	*	*	*	*	*	*	*	*
T.h.+2,4D	ns	*	*		*	ns	*	ns	*	ns	*	ns
F.c.	*	*	*	*		*	*	*	*	*	ns	*
F.c.+2,4D	ns	*	*	ns	*		*	ns	*	ns	*	ns
T.h.+F.c.	*	ns	*	*	*	*		*	ns	*	*	*
T.h.+F.c.+2,4D	ns	*	*	ns	*	ns	*		*	ns	*	ns
ex.T.h.	*	ns	*	*	*	*	ns	*		*	*	*
ex.T.h.+2,4D	ns	*	*	ns	*	ns	*	ns	*		*	ns
ex.T.h.+F.c.	*	*	*	*	ns	*	*	*	*	*		*
ex.T.h.+F.c.+2,4D	ns	*	*	ns	*	ns	*	ns	*	ns	*	

Table S2. Comparison of ANOVA Tukey post-hoc results for seeds germination with treatment as a factor, (T.h. – *Trichoderma harzianum*, F.c.- *Fusarium culmorum,* ex. T.h – *T. harzianum* extracellular metabolites treated wheat seeds), p significance was shown: * - p ≤ 0.001, ns – not significant.

ANOVA result for germination

	Control	2,4D	T.h.	T.h.+2,4D	F.c.	F.c.+2,4D	T.h.+F.c.	T.h.+F.c.+2,4D	ex.T.h.	ex.T.h.+2,4D	ex.T.h.+F.c.	ex.T.h.+F.c.+2,4D
Control		ns	ns	ns	*	*	*	*	ns	ns	*	*
2,4D	ns		ns	ns	*	*	*	*	ns	ns	*	*
T.h.	ns	ns		ns	*	*	*	*	ns	ns	*	*
T.h.+2,4D	ns	ns	ns		*	*	*	*	ns	ns	*	*
F.c.	*	*	*	*		ns	ns	ns	*	*	ns	ns
F.c.+2,4D	*	*	*	*	ns		ns	ns	*	*	ns	ns
T.h.+F.c.	*	*	*	*	ns	ns		ns	*	*	ns	ns
T.h.+F.c.+2,4D	*	*	*	*	ns	ns	ns		*	*	ns	ns
ex.T.h.	ns	ns	ns	ns	*	*	*	*		ns	*	*
ex.T.h.+2,4D	ns	ns	ns	ns	*	*	*	*	ns		*	*
ex.T.h.+F.c.	*	*	*	*	ns	ns	ns	ns	*	*		ns
ex.T.h.+F.c.+2,4D	*	*	*	*	ns	ns	ns	ns	*	*	ns	

Table S3. Comparison of ANOVA Tukey post-hoc results for RWC with treatment as a factor, (T.h. – *Trichoderma harzianum,* F.c.- *Fusarium culmorum,* ex. T.h – *T. harzianum* extracellular metabolites treated wheat seeds), p significance was shown* p ≤ 0.001, ** p ≤ 0.0001, ns – not significant.

ANOVA result for RWC

	Control	2,4D	T.h.	T.h.+2,4D	F.c.	F.c.+2,4D	T.h.+F.c.	T.h.+F.c.+2,4D	ex.T.h.	ex.T.h.+2,4D	ex.T.h.+F.c.	ex.T.h.+F.c.+2, 4D
Control		*	ns	ns	**	**	**	**	ns	*	**	**
2,4D	*		ns	ns	**	**	ns	**	*	ns	ns	*
T.h.	ns	ns		ns	**	**	**	**	ns	ns	*	**
T.h.+2,4D	ns	ns	ns		**	**	**	**	ns	ns	ns	**
F.c.	**	**	**	**		ns	**	ns	**	**	**	**
F.c.+2,4D	**	**	**	**	ns		**	ns	**	**	**	**
T.h.+F.c.	**	ns	**	**	**	**		**	**	ns	ns	ns
T.h.+F.c.+2,4D	**	**	**	**	ns	ns	**		**	**	**	**
ex.T.h.	ns	*	ns	ns	**	**	**	**		ns	*	**
ex.T.h.+2,4D	*	ns	ns	ns	**	**	ns	**	ns		ns	*
ex.T.h.+F.c.	**	ns	*	ns	**	**	ns	**	*	ns		ns
ex.T.h.+F.c.+2, 4D	**	*	**	**	**	**	ns	**	**	*	ns	

Table S4. Comparison of ANOVA Tukey post-hoc results for chlorophyll content with treatment as a factor, (T.h. – *Trichoderma harzianum,* F.c.- *Fusarium culmorum,* ex. T.h – *T. harzianum* extracellular metabolites treated wheat seeds), p significance was shown: * p ≤ 0.001, ** p ≤ 0.0001, ns – not significant.

ANOVA for chlorophyll

	Control	2,4D	T.h.	T.h.+2,4D	F.c.	F.c.+2,4D	T.h.+F.c.	T.h.+F.c.+2,4D	ex.T.h.	ex.T.h.+2,4D	ex.T.h.+F.c.	ex.T.h.+F.c.+2,4D
Control		*	*	*	*	*	*	*	*	*	*	*
2,4D	*		*	*	*	*	*	*	*	*	*	*
T.h.	*	*		*	*	*	*	*	*	*	*	*
T.h.+2,4D	*	*	*		*	*	ns	ns	ns	*	*	*
F.c.	*	*	*	*		*	*	*	*	*	*	*
F.c.+2,4D	*	*	*	*	*		*	*	*	*	*	*
T.h.+F.c.	*	*	*	ns	*	*		ns	ns	*	*	*
T.h.+F.c.+2,4D	*	*	*	ns	*	*	ns		*	*	*	*
ex.T.h.	*	*	*	ns	*	*	ns	*		*	*	*
ex.T.h.+2,4D	*	*	*	*	*	*	*	*	*		*	*
ex.T.h.+F.c.	*	*	*	*	*	*	*	*	*	*		*
ex.T.h.+F.c.+2,4D	*	*	*	*	*	*	*	*	*	*	*	

Table S5. Comparison of ANOVA Tukey post-hoc results for JA with treatment as a factor, (T.h. – *Trichoderma harzianum,* F.c.- *Fusarium culmorum,* ex. T.h – *T. harzianum* extracellular metabolites treated wheat seeds), p significance was shown: ** - $p \leq 0.001$, * - $p \leq 0.01$, ns – not significant.

	**ANOVA result for JA**											
	Control	2,4D	T.h.	T.h.+2,4D	F.c.	F.c.+2,4D	T.h.+F.c.	T.h.+F.c.+2,4D	ex.T.h.	ex.T.h.+2,4D	ex.T.h.+F.c.	ex.T.h.+F.c.+2,4 D
Control		**	ns	**	**	**	**	**	*	**	**	**
2,4D	**		**	*	**	**	**	*	**	**	**	**
T.h.	ns	**		**	**	**	**	**	**	**	**	**
T.h.+2,4D	**	*	**		**	**	**	ns	**	**	**	ns
F.c.	**	**	**	**		**	ns	**	**	**	**	**
F.c.+2,4D	**	**	**	**	**		**	**	**	ns	*	**
T.h.+F.c.	**	**	**	**	ns	**		**	**	**	**	**
T.h.+F.c.+2,4D	**	*	**	ns	**	**	**		**	**	**	*
ex.T.h.	*	**	*	**	**	**	**	**		**	**	**
ex.T.h.+2,4D	**	**	**	**	**	ns	**	**	**		**	**
ex.T.h.+F.c.	**	ns	**	**	**	*	**	**	**	**		ns
ex.T.h.+F.c.+2,4 D	**	ns	**	ns	**	**	**	*	**	**	ns	

Table S6. Comparison of ANOVA Tukey post-hoc results for oxylipin with treatment as a factor, (T.h. – *Trichoderma harzianum,* F.c.- *Fusarium culmorum,* ex. T.h – *T. harzianum* extracellular metabolites treated wheat seeds), p significance was shown: ** - p ≤ 0.001, * - p ≤ 0.01, ns – not significant

9-Hotre (shoot)

	Control	2,4D	T.h.	T.h.+2,4D	F.c.	F.c.+2,4D	T.h.+F.c.	T.h.+F.c.+2,4D	ex.T.h.	ex.T.h.+2,4D	ex.T.h.+F.c.	ex.T.h.+F.c.+2,4D
Control		*	*	*	*	ns	*	*	*	*	*	*
2,4D	*		*	ns	*	*	ns	*	*	*	*	*
T.h.	*	*		*	*	*	*	*	ns	*	*	*
T.h.+2,4D	*	ns	*		*	*	ns	*	*	*	*	*
F.c.	*	*	*	*		*	ns	*	*	ns	*	*
F.c.+2,4D	ns	*	*	*	*		*	*	*	*	*	*
T.h.+F.c.	*	ns	*	ns	ns	*		*	*	*	*	*
T.h.+F.c.+2,4D	*	*	*	*	*	*	*		ns	*	*	*
ex.T.h.	*	*	ns	*	*	*	*	ns		*	*	*
ex.T.h.+2,4D	*	*	*	*	ns	*	*	*	*		*	*
ex.T.h.+F.c.	*	*	*	*	*	*	*	*	*	*		ns
ex.T.h.+F.c.+2,4D	*	*	*	*	*	*	*	*	*	*	ns	

13-HODE (shoot)

	Control	2,4D	T.h.	T.h.+2,4D	F.c.	F.c.+2,4D	T.h.+F.c.	T.h.+F.c.+2,4D	ex.T.h.	ex.T.h.+2,4D	ex.T.h.+F.c.	ex.T.h.+F.c.+2,4D
Control		*	*	*	*	ns	ns	*	*	ns	*	*
2,4D	*		*	*	*	*	*	*	*	*	*	ns
T.h.	*	*		ns	*	*	*	*	ns	*	*	*
T.h.+2,4D	*	*	ns		*	*	*	ns	ns	*	*	*
F.c.	*	*	*	*		*	*	*	*	*	*	*
F.c.+2,4D	ns	*	*	*	*		ns	*	*	ns	ns	*
T.h.+F.c.	ns	*	*	*	*	ns		*	*	ns	ns	*
T.h.+F.c.+2,4D	*	*	*	ns	*	*	*		ns	*	*	*
ex.T.h.	*	*	ns	ns	*	*	*	ns		*	*	*
ex.T.h.+2,4D	ns	*	*	*	*	ns	ns	*	*		ns	*
ex.T.h.+F.c.	*	*	*	*	*	ns	ns	*	*	ns		*

ex.T.h.+F.c.+2,4D	*	ns	*	*	*	*	*	*	*	*	*	

9-HODE (shoot)

	Control	2,4D	T.h.	T.h.+2,4D	F.c.	F.c.+2,4D	T.h.+F.c.	T.h.+F.c.+2,4D	ex.T.h.	ex.T.h.+2,4D	ex.T.h.+F.c.	ex.T.h.+F.c.+2,4D
Control		*	*	*	*	*	*	*	*	*	*	ns
2,4D	*		*	*	*	*	*	*	*	*	*	*
T.h.	*	*		ns	*	*	*	ns	ns	*	ns	*
T.h.+2,4D	*	*	ns		*	ns	*	ns	ns	*	ns	*
F.c.	*	*	*	*		*	*	*	*	*	*	*
F.c.+2,4D	*	*	*	ns	*		*	ns	ns	ns	ns	*
T.h.+F.c.	*	*	*	*	*	*		*	*	ns	*	*
T.h.+F.c.+2,4D	*	*	ns	ns	*	ns	*		ns	*	ns	*
ex.T.h.	*	*	ns	ns	*	ns	*	ns		*	ns	*
ex.T.h.+2,4D	*	*	*	*	*	ns	ns	*	*		*	*
ex.T.h.+F.c.	*	*	ns	ns	*	ns	*	ns	ns	*		*
ex.T.h.+F.c.+2,4D	ns	*	*	*	*	*	*	*	*	*	*	

13-Hotre (shoot)

	Control	2,4D	T.h.	T.h.+2,4D	F.c.	F.c.+2,4D	T.h.+F.c.	T.h.+F.c.+2,4D	ex.T.h.	ex.T.h.+2,4D	ex.T.h.+F.c.	ex.T.h.+F.c.+2,4D
Control		ns	*	ns	*	ns	*	*	*	*	*	ns
2,4D	ns		*	ns	*	ns	*	*	*	*	*	ns
T.h.	*	*		*	*	ns	ns	ns	*	ns	ns	*
T.h.+2,4D	ns	ns	*		*	ns	*	*	*	*	*	ns
F.c.	*	*	*	*		*	*	*	*	*	*	*
F.c.+2,4D	ns	ns	ns	ns	*		*	*	*	*	*	ns
T.h.+F.c.	*	*	ns	*	*	*		ns	ns	ns	ns	*
T.h.+F.c.+2,4D	*	*	ns	*	*	*	ns		*	ns	ns	*
ex.T.h.	*	*	*	*	*	*	ns	*		ns	ns	*
ex.T.h.+2,4D	*	*	ns	*	*	*	ns	ns	ns		ns	*
ex.T.h.+F.c.	*	*	ns	*	*	*	ns	ns	ns	ns		*
ex.T.h.+F.c.+2,4D	ns	ns	*	ns	*	ns	*	*	*	*	*	

9Hotre (root)

	Control	2,4D	T.h.	T.h.+2,4D	F.c.	F.c.+2,4D	T.h.+F.c.	T.h.+F.c.+2,4D	ex.T.h.	ex.T.h.+2,4D	ex.T.h.+F.c.	ex.T.h.+F.c.+2,4 D
Control		*	*	ns	*	ns	*	*	*	*	*	*
2,4D	*		*	ns	*	*	*	ns	*	*	*	ns
T.h.	*	*		*	*	*	*	*	*	*	*	*
T.h.+2,4D	ns	ns	*		*	ns	*	ns	*	*	*	ns
F.c.	*	*	*	*		*	*	*	ns	*	*	*
F.c.+2,4D	ns	*	*	ns	*		*	*	*	*	*	*
T.h.+F.c.	*	*	*	*	*	*		*	*	*	*	*
T.h.+F.c.+2,4D	*	ns	*	ns	*	*	*		*	*	*	ns
ex.T.h.	*	*	*	*	ns	*	*	*		*	*	*
ex.T.h.+2,4D	*	*	*	*	*	*	*	*	*		ns	ns
ex.T.h.+F.c.	*	*	*	*	*	*	*	*	*	ns		*
ex.T.h.+F.c.+2,4 D	*	ns	*	ns	*	*	*	ns	*	ns	*	

13Hode (root)

	Control	2,4D	T.h.	T.h.+2,4D	F.c.	F.c.+2,4D	T.h.+F.c.	T.h.+F.c.+2,4D	ex.T.h.	ex.T.h.+2,4D	ex.T.h.+F.c.	ex.T.h.+F.c.+2,4 D
Control		*	*	*	*	ns	*	*	*	ns	*	*
2,4D	*		*	*	ns	*	*	ns	*	*	*	*
T.h.	*	*		*	*	*	*	*	*	*	*	*
T.h.+2,4D	*	*	*		*	*	*	*	*	*	ns	ns
F.c.	*	ns	*	*		*	*	ns	*	ns	*	*
F.c.+2,4D	ns	*	*	*	*		*	*	*	ns	*	*
T.h.+F.c.	*	*	*	*	*	*		*	ns	*	*	*
T.h.+F.c.+2,4D	*	ns	*	*	ns	*	*		*	ns	*	*
ex.T.h.	*	*	*	*	*	*	ns	*		*	*	*
ex.T.h.+2,4D	ns	*	*	*	ns	ns	*	ns	*		*	*
ex.T.h.+F.c.	*	*	*	ns	*	*	*	*	*	*		*
ex.T.h.+F.c.+2,4 D	*	*	*	ns	*	*	*	*	*	*	*	

9Hode (root)

	Control	2,4D	T.h.	T.h.+2,4D	F.c.	F.c.+2,4D	T.h.+F.c.	T.h.+F.c.+2,4D	ex.T.h.	ex.T.h.+2,4D	ex.T.h.+F.c.	ex.T.h.+F.c.+2,4D
Control		*	*	*	*	*	*	*	*	*	ns	*
2,4D	*		ns	ns	ns	ns	*	ns	ns	ns	*	ns
T.h.	*	ns		ns	*	ns	*	ns	ns	ns	ns	ns
T.h.+2,4D	*	ns	ns		*	ns	*	ns	ns	ns	ns	ns
F.c.	*	ns	*	*		ns	ns	ns	*	*	*	*
F.c.+2,4D	*	ns	ns	ns	ns		ns	ns	ns	ns	*	ns
T.h.+F.c.	*	*	*	*	ns	ns		ns	*	*	*	*
T.h.+F.c.+2,4D	*	ns	ns	ns	ns	ns	ns		ns	*	*	ns
ex.T.h.	*	ns	ns	ns	*	ns	*	ns		ns	ns	ns
ex.T.h.+2,4D	*	ns	ns	ns	*	ns	*	*	ns		ns	ns
ex.T.h.+F.c.	ns	*	ns	ns	*	*	*	*	ns	ns		ns
ex.T.h.+F.c.+2,4D	*	ns	ns	ns	*	ns	*	ns	ns	ns	ns	

13Hotre (root)

	Control	2,4D	T.h.	T.h.+2,4D	F.c.	F.c.+2,4D	T.h.+F.c.	T.h.+F.c.+2,4D	ex.T.h.	ex.T.h.+2,4D	ex.T.h.+F.c.	ex.T.h.+F.c.+2,4D
Control		*	*	*	*	*	*	*	*	*	*	*
2,4D	*		*	ns	ns	ns	*	ns	*	*	*	*
T.h.	*	*		*	*	*	*	*	*	*	*	*
T.h.+2,4D	*	ns	*		ns	ns	*	*	*	*	*	*
F.c.	*	ns	*	ns		ns	*	*	*	*	*	*
F.c.+2,4D	*	ns	*	ns	ns		*	*	*	*	*	*
T.h.+F.c.	*	*	*	*	*	*		*	*	*	*	*
T.h.+F.c.+2,4D	*	ns	*	*	*	*	*		*	*	*	*
ex.T.h.	*	*	*	*	*	*	*	*		*	*	*
ex.T.h.+2,4D	*	*	*	*	*	*	*	*	*		*	*
ex.T.h.+F.c.	*	*	*	*	*	*	*	*	*	*		*
ex.T.h.+F.c.+2,4D	*	*	*	*	*	*	*	*	*	*	*	

Table S7. Comparison of ANOVA Tukey post-hoc results for CAT and SOD with treatment as a factor, (T.h. – *Trichoderma harzianum,* F.c.- *Fusarium culmorum,* ex. T.h – *T. harzianum* extracellular metabolites treated wheat seeds), p significance was shown: * - p ≤0.05 ** - p ≤0.01, *** - p ≤0.001, ns – not significant.

ANOVA for CAT (shoot)

	Control	2,4-D	T.h	T.h 2,4-D	F.c.	F.c. 2,4-D	T.h. F.c.	T.h. F.c. 2,4-D	e.T.h.	e.T.h. 2,4-D	e.T.h. F.c.	e.T.h. F.c. 2,4-D
Control		**	ns	*	***	***	***	***	***	***	***	***
2,4-D	**		ns	ns	***	***	ns	***	ns	ns	ns	***
T.h	ns	ns		ns	***	***	***	***	**	**	*	***
T.h 2,4-D	*	ns	ns		***	***	***	***	**	**	ns	***
F.c.	***	***	***	***		ns	ns	***	**	*	**	ns
F.c. 2,4-D	***	***	***	***	ns		***	*	***	***	***	ns
T.h. F.c.	***	ns	***	***	ns	***		***	ns	ns	ns	***
T.h. F.c. 2,4-D	***	***	***	***	***	*	***		***	***	***	ns
e.T.h.	***	ns	**	**	**	***	ns	***		ns	ns	***
e.T.h. 2,4-D	***	ns	**	**	*	***	ns	***	ns		ns	***
e.T.h. F.c.	***	ns	*	ns	**	***	ns	***	ns	ns		***
e.T.h. F.c. 2,4-D	***	***	***	***	ns	ns	***	ns	***	***	***	

ANOVA for CAT (root)

	Control	2,4-D	T.h	T.h 2,4-D	F.c.	F.c. 2,4-D	T.h. F.c.	T.h. F.c. 2,4-D	e.T.h.	e.T.h. 2,4-D	e.T.h. F.c.	e.T.h. F.c. 2,4-D
Control		ns	ns	ns	ns	***	ns	***	ns	*	ns	ns
2,4-D	ns		ns	ns	ns	***	ns	**	ns	ns	ns	ns
T.h	ns	ns		ns	ns	***	ns	**	ns	ns	ns	ns
T.h 2,4-D	ns	ns	ns		ns	***	ns	***	ns	*	ns	ns
F.c.	ns	ns	ns	ns		***	ns	*	ns	ns	ns	ns
F.c. 2,4-D	***	***	***	***	***		***	ns	**	*	***	***
T.h. F.c.	ns	ns	ns	ns	ns	***		***	ns	**	ns	*
T.h. F.c. 2,4-D	***	**	**	***	*	ns	***		**	ns	***	ns

e.T.h.	ns	ns	ns	ns	ns	***	ns	**		ns	ns	ns
e.T.h. 2,4-D	*	ns	ns	*	ns	*	**	ns	ns		ns	ns
e.T.h. F.c.	ns	ns	ns	ns	ns	***	ns	**	ns	ns		ns
e.T.h. F.c. 2,4-D	ns	ns	ns	ns	ns	**	*	ns	ns	ns	ns	

ANOVA for SOD (shoot)

	Control	2,4-D	T.h	T.h 2,4-D	F.c.	F.c. 2,4-D	T.h. F.c.	T.h. F.c. 2,4-D	e.T.h.	e.T.h. 2,4-D	e.T.h. F.c.	e.T.h. F.c. 2,4-D
Control		ns	ns	ns	ns	***	ns	ns	ns	ns	ns	***
2,4-D	ns		ns	ns	ns	***	*	ns	ns	ns	ns	***
T.h	ns	ns		ns	ns	***	ns	ns	*	ns	ns	***
T.h 2,4-D	ns	ns	ns		ns	***	ns	ns	*	ns	ns	***
F.c.	ns	ns	ns	ns		***	ns	ns	ns	ns	ns	***
F.c. 2,4-D	***	***	***	***	***		***	***	**	***	***	ns
T.h. F.c.	ns	*	ns	ns	ns	***		ns	**	ns	ns	***
T.h. F.c. 2,4-D	ns	ns	ns	ns	ns	***	ns		ns	ns	ns	***
e.T.h.	ns	ns	*	ns	ns	**	**	ns		ns	ns	***
e.T.h. 2,4-D	ns	ns	ns	ns	ns	***	ns	ns	ns		ns	***
e.T.h. F.c.	ns	ns	ns	ns	ns	***	ns	ns	ns	ns		***
e.T.h. F.c. 2,4-D	***	***	***	***	***	ns	***	***	***	***	***	

ANOVA for SOD (root)

	Control	2,4-D	T.h	T.h 2,4-D	F.c.	F.c. 2,4-D	T.h. F.c.	T.h. F.c. 2,4-D	e.T.h.	e.T.h. 2,4-D	e.T.h. F.c.	e.T.h. F.c. 2,4-D
Control		ns	***	**	***	***	***	ns	ns	**	***	ns
2,4-D	ns		ns	ns	***	***	***	***	ns	ns	***	***
T.h	***	ns		ns	***	***	***	***	ns	ns	***	***
T.h 2,4-D	**	ns	ns		***	***	***	***	ns	ns	***	***
F.c.	***	***	***	***		ns	ns	***	***	***	***	***
F.c. 2,4-D	***	***	***	***	ns		***	***	***	***	***	***
T.h. F.c.	***	***	***	***	ns	***		***	***	***	***	***
T.h. F.c. 2,4-D	ns	*	***	***	***	***	***		***	***	***	ns
e.T.h.	ns	ns	ns	ns	***	***	***	***		ns	***	***
e.T.h. 2,4-D	**	ns	ns	ns	***	***	***	***	ns		***	***
e.T.h. F.c.	***	***	***	***	***	***	***	***	***	***		*
e.T.h. F.c. 2,4-D	ns	***	***	***	***	***	***	ns	***	***	*	

Table S8. Comparison of ANOVA Tukey post-hoc results for ZEA with treatment as a factor, (T.h. – *Trichoderma harzianum,* F.c.- *Fusarium culmorum,* ex. T.h – *T. harzianum* extracellular metabolites treated wheat seeds), p significance was shown: * p ≤ 0.01, ** p ≤ 0.001, ns – not significant

ANOVA for ZEA

	F.c.	F.c.+2,4D	T.h.+F.c.	T.h.+F.c.+2,4D	ex.T.h.+F.c.	ex.T.h.+F.c.+2,4D
F.c.		**	*	ns	**	ns
F.c.+2,4D	**		**	**	**	*
T.h.+F.c.	*	**		ns	**	ns
T.h.+F.c.+2,4D	ns	**	ns		**	ns
ex.T.h.+F.c.	**	**	**	**		**
ex.T.h.+F.c.+2,4D	ns	*	ns	ns	**	

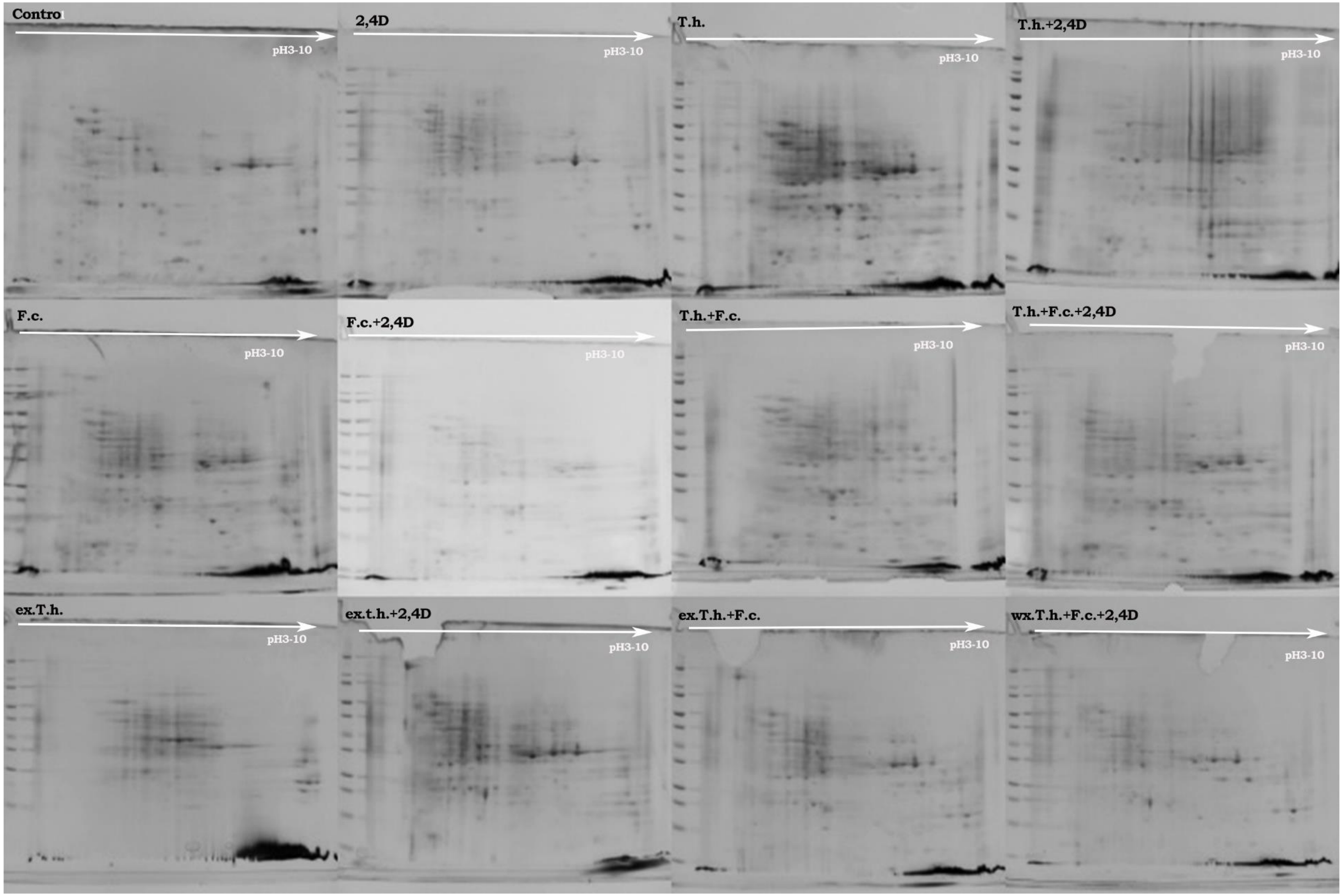
Contro
pH3-10
2,4D
pH3-10
T.h.
pH3-10
T.h.+2,4D
pH3-10
F.c.
pH3-10
F.c.+2,4D
pH3-10
T.h.+F.c.
pH3-10
T.h.+F.c.+2,4D
pH3-10
ex.T.h.
pH3-10
ex.t.h.+2,4D
pH3-10
ex.T.h.+F.c.
pH3-10
wx.T.h.+F.c.+2,4D
pH3-10

Figure S1. 2-D SDS PAGE protein profile from root material (Control; 2,4D; *T. harzianum* inoculated; *T. harzianum* + 2,4D; *F. culmorum* inoculated; *F. culmorum* + 2,4D; *T. harzianum* + *F. culmorum; T. harzianum* + *F. culmorum* + 2,4D; treated with extracellular extract *T. harzianum*; with extracellular extract *T. harzianum* + 2,4D; with extracellular extract *T. harzianum* + *F. culmorum*; extracellular extract *T. harzianum* + *F. culmorum* + 2,4D).

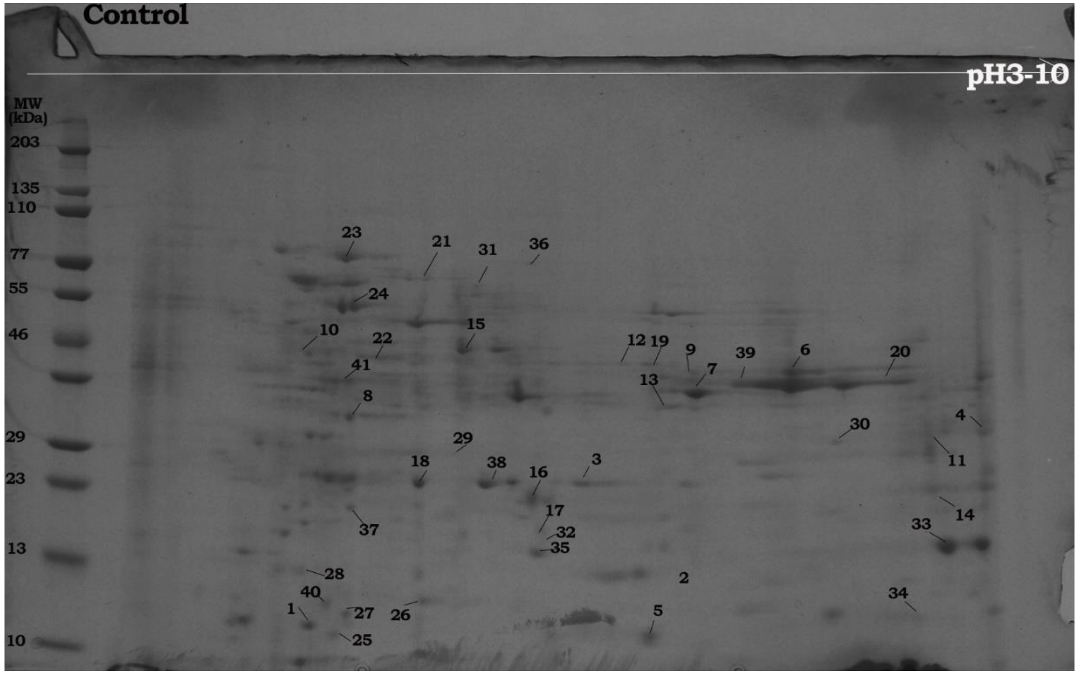

Figure S2. The spots analyzed during the proteomic study from root material, applied to a control gel, as an example.

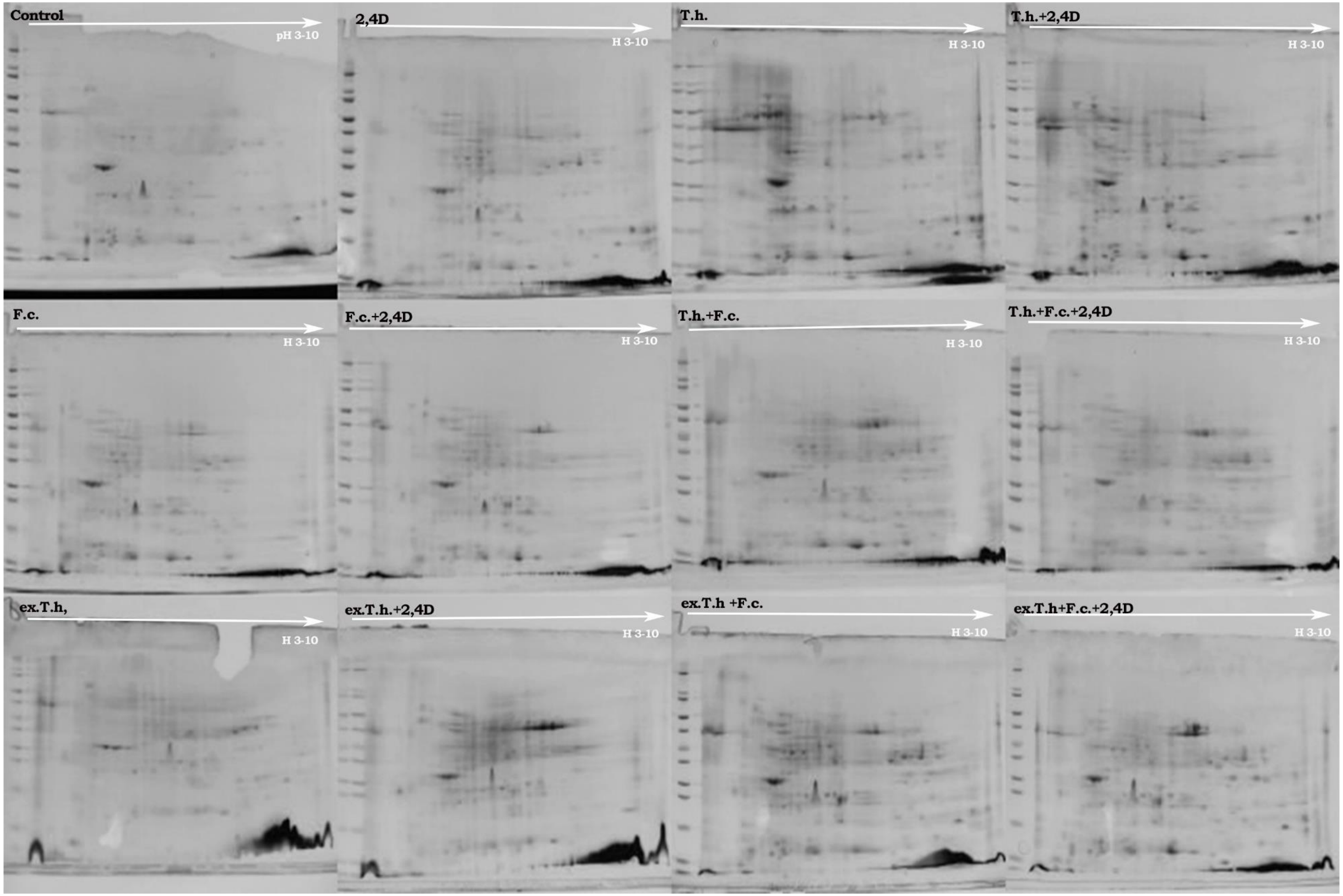

Control
pH 3-10
2,4D
H 3-10
T.h.
H 3-10
T.h.+2,4D
H 3-10
F.c.
H 3-10
F.c.+2,4D
H 3-10
T.h.+F.c.
H 3-10
T.h.+F.c.+2,4D
H 3-10
ex.T.h,
H 3-10
ex.T.h.+2,4D
H 3-10
ex.T.h +F.c.
H 3-10
ex.T.h+F.c.+2,4D
H 3-10

Figure S3. 2-D SDS PAGE protein profile from shoot material (Control; 2,4D; *T. harzianum* inoculated; *T. harzianum* + 2,4D; *F. culmorum* inoculated; *F. culmorum* + 2,4D; *T. harzianum* + *F. culmorum; T. harzianum* + *F. culmorum* + 2,4D; treated with extracellular extract *T. harzianum*; with extracellular extract *T. harzianum* + 2,4D; with extracellular extract *T. harzianum* + *F. culmorum*; extracellular extract *T. harzianum* + *F. culmorum* + 2,4D).

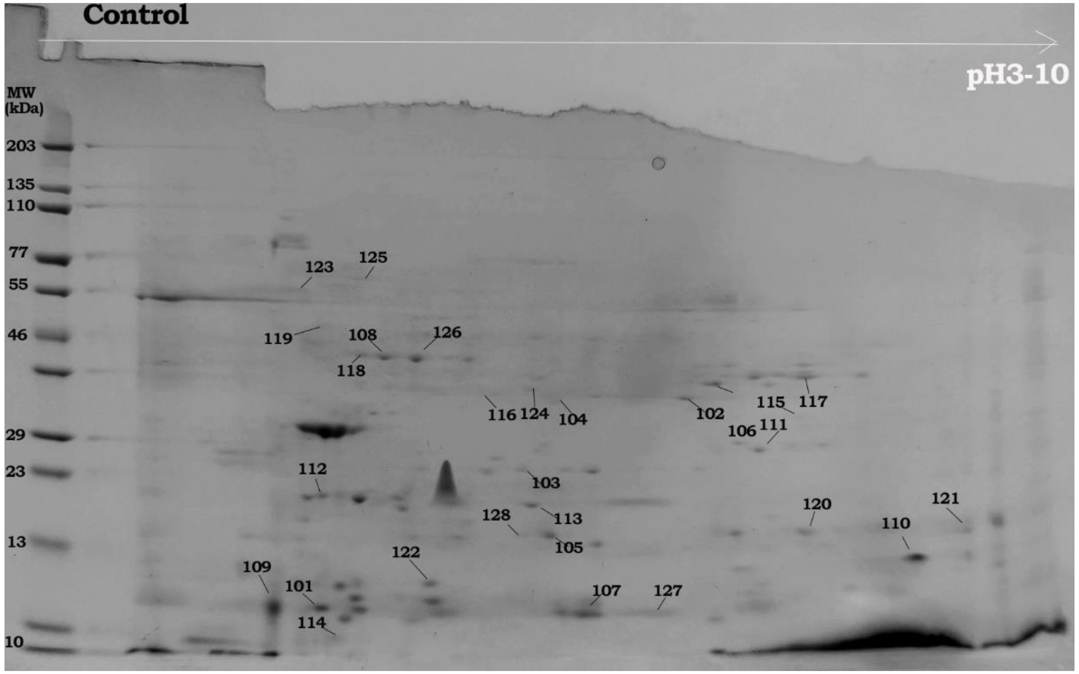

Figure S4. The spots analyzed during the proteomic study from shoot material, applied to a control gel, as an example.
